# Fecal microbiota transplantation confers beneficial metabolic effects of diet and exercise on diet-induced obese mice

**DOI:** 10.1038/s41598-018-33893-y

**Published:** 2018-10-23

**Authors:** Zi-Lun Lai, Ching-Hung Tseng, Hsiu J. Ho, Cynthia K. Y. Cheung, Jian-Yong Lin, Yi-Ju Chen, Fu-Chou Cheng, Yao-Chun Hsu, Jaw-Town Lin, Emad M. El-Omar, Chun-Ying Wu

**Affiliations:** 1 Division of Gastroenterology, Taichung Veterans General Hospital, Taichung, Taiwan; 2 Germark Biotechnology Co., Ltd., Taichung, Taiwan; 3 Institute of Digestive Disease, the Chinese University of, Hong Kong Shatin, Hong Kong; 4 Faculty of Medicine and Graduate Institute of Clinical Medicine, National Yang-Ming University, Taipei, Taiwan; 5 Department of Dermatology, Taichung Veterans General Hospital, Taichung, Taiwan; 6 Stem Cell Center, Department of Medical Research, Taichung Veterans General Hospital, Taichung, Taiwan; 7 Department of Internal Medicine, E-Da Hospital/I-Shou University, Kaohsiung, Taiwan; 8 Graduate Institute of Clinical Medicine, China Medical University, Taichung, Taiwan; 9 School of Medicine, Fu Jen Catholic University, New Taipei City, Taiwan; 10 Institute of Population Health Sciences, National Health Research Institutes, Miaoli, Taiwan; 11 Microbiome Research Centre, St George and Sutherland Clinical School, University of New South Wales, Sydney, Australia; 12 Division of Translational Research, Taipei Veterans General Hospital, Taipei, Taiwan; 13 National Institute of Cancer Research, National Health Research Institutes, Miaoli, Taiwan; 14 Department of Public Health, China Medical University, Taichung, Taiwan; 15 Department of Life Sciences and Rong Hsing Research Center for Translational Medicine, National Chung-Hsing University, Taichung, Taiwan

## Abstract

Diet and exercise are conventional methods for controlling body weight and are linked to alterations in gut microbiota. However, the associations of diet, exercise, and gut microbiota in the control of obesity remain largely unknown. In the present study, using 16S rRNA amplicon sequencing and fecal microbiota transplantation (FMT), normal fat diet (NFD), exercise and their combination resulted in improved metabolic profiles in comparison to sedentary lifestyle with high fat diet (HFD). Moreover, diet exerted more influence than exercise in shaping the gut microbiota. HFD-fed mice receiving FMT from NFD-exercised donors not only showed remarkably reduced food efficacy, but also mitigated metabolic profiles (*p* < 0.05). The transmissible beneficial effects of FMT were associated with bacterial genera *Helicobacter*, *Odoribacter* and AF12 and overrepresentation of oxidative phosphorylation and glycolysis genes. Our findings demonstrate that the beneficial effects of diet and exercise are transmissible via FMT, suggesting a potential therapeutic treatment for obesity.

## Introduction

The gut microbiota plays an important role in human metabolism, and an imbalance in its composition (i.e., dysbiosis) has been associated with obesity and other metabolic abnormalities. It is influenced by several factors and its modification can alter host adiposity via different mechanisms^1,2^. In keeping with empirical evidence that normal diet and exercise are beneficial to human health, especially in terms of weight control, emerging data have linked dietary interventions and exercise to altered gut microbiota in obese mice^3,4^ and professional athletes^5^, suggesting that gut microbiota mediates the beneficial physiological outcomes of diet and exercise.

Gut microbiota resulting from normal diet, in which there is reduced caloric intake, has been reported to ameliorate metabolic disturbance in murine models with obesity^3,6,7^. Microbial mechanisms underlying the detrimental effects of high fat diet (HFD) have also been postulated largely in mouse models. At first, HFD causes microbial dysbiosis in the gut (for example, by promoting the growth of opportunistic pathogens^8^), altering intestinal integrity^9,10^ (i.e., leaky gut), which results in endotoxemia and ultimately obesity^11^. The association between HFD and endotoxemia was also shown in healthy human subjects^12,13^. Previous studies have sequentially linked HFD to obesity through the effects of the gut microbiota.

In mouse models, exercise increases caloric consumption and alters the gut bacterial community, attenuating some obesity-related changes^4,14^. These results shed new light on obesity management. In addition to the established hormonal alterations in humans (e.g., peptide YY^15^ and ghrelin^16^) that reduce appetite, exercise in rats supposedly alters gut microbiota and increases butyrate concentration in the cecum^17^. In humans, butyrate is known to reduce inflammation through NF-κB inhibition^18^, and its beneficial effects on host insulin sensitivity^19^ and immunity^20^ were shown in mice. Several mechanisms by which exercise modifies gut microbiota have been proposed. For example, exercise-induced stress activates the hypothalamic-pituitary-adrenal axis and causes the release of various hormones (e.g., corticotropin, noradrenaline, and serotonin), which impact the gastrointestinal environment and alter the gut microbiota^21^. Exercise also reduces human gut transit time^22^ and changes stool consistency, which has been shown to be strongly associated with human gut microbial composition^23,24^. Although prolonged vigorous exercise can cause impaired intestinal permeability^25^, highly trained athletes have been found to have lower levels of blood endotoxin^26^, suggesting that chronic physical training improves gut barrier integrity.

Despite the myriad of studies on the beneficial effects of normal diet and exercise on obesity and corresponding alterations in the gut microbiota, little is known about their joint effects in the control of obesity. In addition, whether fecal microbiota transplantation (FMT) can transmit the beneficial effects of diet and exercise to alter metabolic profiles has not been investigated.

Here, we show that the weight reducing effects of normal diet and exercise, and related improvements in metabolic and inflammatory profiles, are transmissible via FMT. Our findings highlight the critical role of the gut microbiota in obesity and suggest that FMT from donors with balanced diet or regular workout (or in combination) is of potential benefit to recipients in terms of altering metabolic and inflammatory profiles.

## Results

**Figure 1:**
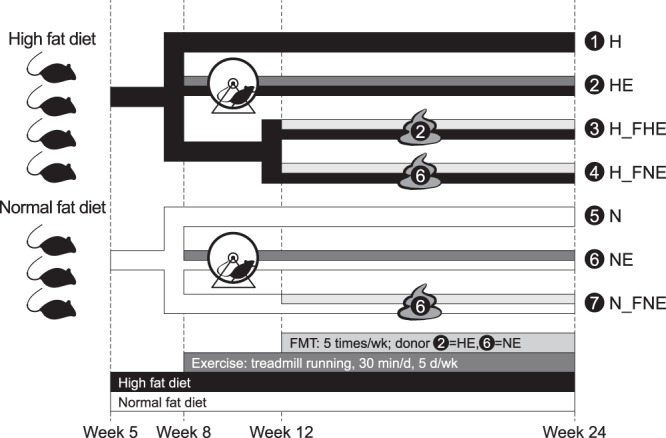
Schematic representation of study design. Seven groups of mice were defined in this study. Abbreviations indicate treatments: H, sedentary mice fed high fat diet (*n* = 6); HE, exercised H mice (*n* = 7); N, sedentary mice fed normal fat diet (*n* = 7); NE, exercised N mice (*n* = 6); H_FHE: H mice receiving FMT from HE (*n* = 7); H_FNE, H mice receiving FMT from NE (*n* = 7); N_FNE: N mice receiving FMT from NE (*n* = 7). Icon with number indicates FMT donor group; 2 refers to HE and 6 refers to NE.

According to diet type, exercise and FMT status, mice were divided into seven groups. Treatment naïve groups included H and N, representing mice fed with high fat diet (HFD) and normal fat diet (NFD), respectively. HE and NE refer to mice receiving HFD and NFD, respectively, and undergoing exercise regimen. H_FHE group consisted of H mice receiving FMT by gavage from HE donor and H_FNE and N_FNE groups consisted of H and N mice, respecitvely, receiving FMT from NE donor (Fig. 1). Details of study design and animal model are presented in the Methods section.

### Diet and exercise altered mice metabolism

**Figure 2:**
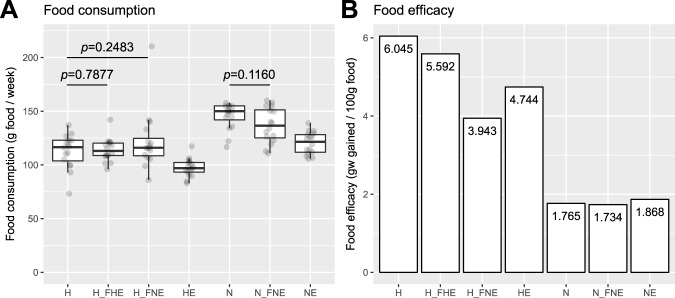
Food consumption and efficacy of each group. (**A**) Food consumption per group was recorded weekly. Data were adjusted to *n* = 7 and compared between non-FMT and FMT recipient groups and *p* values (*t*-test) were obtained. (**B**) Food efficacy of each group was estimated as grams of body weight gain per 100 g food consumed. Data are shown in the figure.

There were no significant differences in food consumption between FMT recipients (i.e., H_FHE, H_FNE and N_FNE) (*p* > 0.05, *t*-test) and their non-FMT counterparts fed the same diet (Fig. 2A), which suggested that gavage merely affects recipients’ feeding behavior. Reductions in food efficacy (i.e., grams of body weight gain per 100 g food consumed) were greatest in H_FNE (34.8% compared to H) followed by HE (21.5%) among mice fed HFD and were similar among NFD-fed mice (Fig. 2B).

The FMT recipient mice underwent a two-day antibiotics regimen before the experiment started (see Methods) to ensure that the outcomes are compatible with clinical guidelines for FMT in humans^27^. As antibiotics increase host susceptibility to obesity^28^, to verify this effect, a supplementary experiment that compared body weight gain and IPGTT results of HFD-fed mice with and without antibiotics exposure was performed (Supplementary file: Supplementary Note 1). There was no significant difference in body weight gain, but glucose tolerance increased significantly (*p* = 0.023, Student’s *t*-test) in HFD-fed mice with antibiotics exposure (Supplementary file: Supplementary Fig. 1, Supplementary Tables 1 and 2), indicating that the FMT recipient mice herein are more susceptible to obesity phenotypes compared to those not exposed to antibiotics.

**Figure 3:**
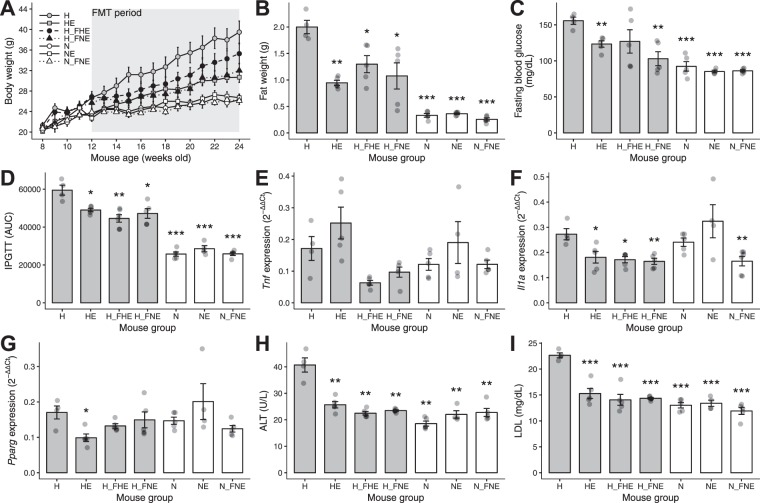
Physiological parameters and inflammatory gene expressions in mice. (**A**) Body weights of all mice involved in the experiment were measured weekly. Data are expressed as mean ± SE per group. (**B**–**I**) Metabolic and inflammatory parameters are expressed as 5% truncated means (data within 5–95% quantile) ± SE. *p < 0.05, **p < 0.01, ***p < 0.001 (Student’s *t*-test, vs. H). Data points are shown in semi-transparency. IPGTT was measured monthly and the measurement at the end of experiment is presented. Other parameters and gene expression levels were determined after mice were euthanized. IPGTT, intraperitoneal glucose tolerance test; *Tnf*, tumor necrosis factor gene; *Il1a*, interleukin 1 alpha gene; *Pparg*, peroxisome proliferator-activated receptor gamma gene; ALT, alanine aminotransferase; LDL, low density lipoprotein.

With regard to the body weight, there were significant differences among groups (*p* < 2 × 10^−16^, two-way ANOVA). H_FNE mice exhibited parallel weight reduction to HE mice (Fig. 3A), in comparison with H group. To better delineate the central tendency of the data, metabolic parameters and gene expression data were compared using a 5%-truncated mean (see Methods). Compared with H mice, N, HE and H_FNE groups demonstrated significant reduction in fat weight, as well as in blood parameters including fasting blood glucose, IPGTT, ALT and LDL (Fig. 3B–I). HE, H_FHE, H_FNE and N_FNE mice also demonstrated lower *Il1a* expressions compared with H mice, while only HE mice showed a lower *Pparg* expression (Fig. 3F–G). Detailed data are shown in Supplementary Table 3 (Supplementary file), and IPGTT curves for each group are in Supplementary Fig. 2 (Supplementary file).

To confirm the effectiveness of HFD, which is associated with lipid accumulation in liver^29,30^, mice liver and fat pad tissues were examined by histological staining and compared among the groups. The results demonstrated that fat deposition in liver tissue and adipocyte size in fat pad tissues were reduced in normal diet and exercised groups (Supplementary file: Supplementary Fig. 3).

### Diet and exercise exerted differential effects on gut microbiota

**Figure 4:**
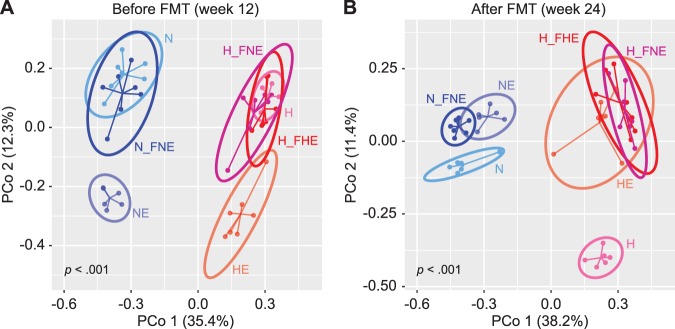
Principal coordinates analysis of mice gut microbiota. PCoA was conducted based on Bray–Curtis distance of operational taxonomic unit (OTU) relative abundance in mice gut microbiota. Groups are distinguished by colors. The significance (*p* value) of between-group inertia was evaluated by Monte-Carlo test (with 1000 permutations). (**A**) PCo ordination of mice gut microbiota before FMT. (**B**) PCo ordination of mice gut microbiota after FMT.

Gut microbial compositions were assessed by fecal 16S ribosomal RNA (rRNA) gene sequencing and statistically analyzed. On principal coordinates analysis (PCoA), the gut microbiota of the groups were separate from each other (*p* < 0.001, Monte-Carlo simulation). Diet was more influential than exercise in shaping the gut microbiota, as demonstrated by greater separation among groups based on diet than among groups based on exercise status (Fig. 4A) and verified by 2-way PERMANONA (diet: *R*^2^ = 0.297, *p* = 0.001; exercise: *R*^2^ = 0.098, *p* = 0.001). A similar outcome was obtained by linear modeling of Shannon index, which showed diet as the most important factor associated with microbiota diversity (*p* = 3.26 × 10^−7^) (Supplementary file: Supplementary Fig. 4).

### Beneficial effects of diet and exercise were transmissible through FMT

To examine whether the effects of normal diet and exercise on host metabolism and inflammation are transmissible, FMT experiments were conducted by dividing the mice into three groups: H_FHE, H_FNE and N_FNE (Fig. 1). Results indicated that FMT from NE group donors (H_FNE) has comparable effect to exercise in reducing body and fat weight in HFD group (Fig. 3A,B). FMT from HE donors (H_FHE) also led to significant weight reduction, but was not as effective as FMT from NE donors. With respect to blood parameters and inflammatory cytokine levels, both H_FHE and H_FNE exhibited improved outcomes comparable to those of HE (Fig. 3C–I).

To validate the interaction between the gut microbiota and their transmissible effect on host metabolism, gut microbial compositions before and after FMT were compared (Fig. 4). Among NFD mice, the gut microbiota of N_FNE group was similar to that of N group before FMT and to that of NE group after FMT. Among HFD mice, the H_FHE and H_FNE gut microbiota overlapped with that of H group before FMT, while they shifted towards HE group after FMT (Fig. 4B). It is interesting to note that the H_FNE group harbored similar microbial composition to the HE group, but not to the NE group, after FMT, suggesting that the beneficial effect of exercise is transmissible via FMT and that colonization of transferred microbes depends on the recipient’s diet.

### Genera associated with exercise and diet in FMT recipients

**Figure 5:**
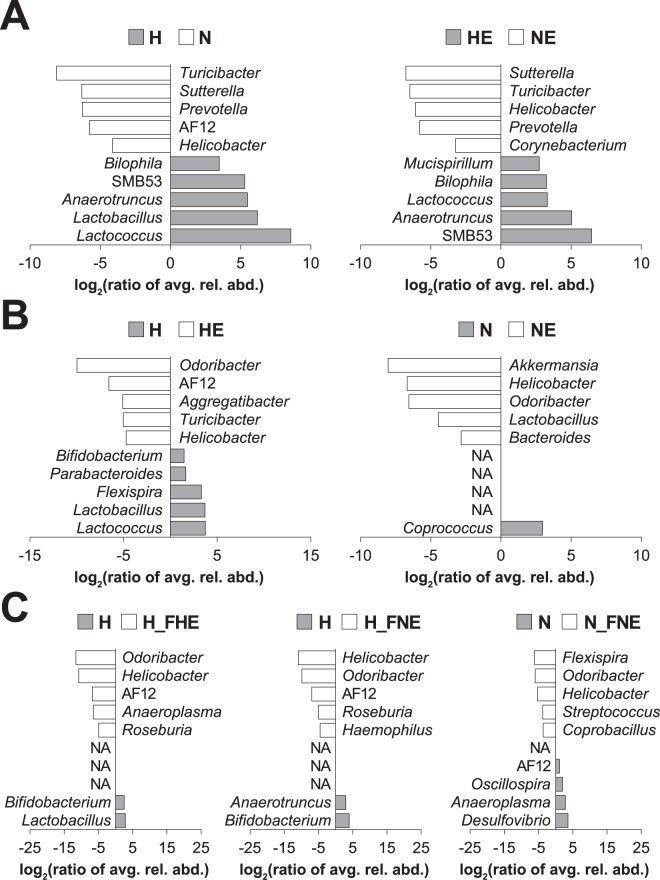
Top genera based on LEfSe and stratified by diet, exercise and FMT. Based on the genera relative abundance after FMT (i.e., week 24), the top 5 genera in each LEfSe comparison (*p* < 0.05) were obtained. The log-2 ratio of genus average relative abundance is presented as x-axis and genus names are presented as inlet. (**A**) Genera in LEfSe comparisons stratified by diet. (**B**) Genera in LEfSe comparisons stratified by exercise. (**C**) Genera in LEfSe comparisons stratified by FMT. NA, not applicable; avg., average; rel., relative; abd., abundance.

The specific alterations in gut microbiota associated with the beneficial effects of diet and exercise were further resolved based on linear discriminant analysis (LDA) effect size (LEfSe). When stratified by diet, *Turicibacter*, *Sutterella*, *Prevotella*, AF12 and *Helicobacter* comprised the top five genera in N and NE groups when compared with H and HE groups (Fig. 5A). When stratified by exercise, *Odoribacter*, AF12, *Helicobacter*, and *Akkermansia* represented the top genera in HE and NE groups when compared with H and N groups (Fig. 5B). *Odoribacter*, *Helicobacter* and AF12 were the top three genera in FMT groups when compared with non-FMT groups (Fig. 5C). The LEfSe results of comparisons among FMT donors and recipients (i.e., HE vs. H_FHE, NE vs. H_FNE and NE vs. F_FNE) are available in Supplementary Fig. 5 (Supplementary file).

### Predicted functional genes associated with exercise

Prompted by the results that both diet and exercise contributed to the microbiota alteration (Fig. 4A) and the well-established sequence of gut microbiota transformed by diet^31^, we investigated the functional genes associated with exercise. As N and NE were shown to share similar phenotypical outcomes (Fig. 3), H and HE mice before FMT were compared to identify those genes differentially enriched by exercise. Among 1250 clusters of orthologous groups (COGs) of significant overabundance (Benjamini-Hochberg adjusted *p* < 0.05) in HE, 93 were related to energy production and 57 to carbohydrate metabolism (Supplementary file: Supplementary Tables 4 and 5), which are functions closely associated with physiological changes induced by exercise.

Genes involved in oxidative phosphorylation were overabundant in the HE gut microbiota, particularly protein complexes in electron transport chain (Supplementary file: Supplementary Table 4) such as Na^+^-transporting NADH:ubiquinone oxidoreductase (COG1347, COG1726, COG1805, COG2209, COG2869 and COG2871), NADH dehydrogenase (COG1252), NADH:ubiquinone oxidoreductase (COG0377, COG0649, COG0713, COG0838, COG0839, COG1005, COG1007 and COG1008), succinate dehydrogenase (COG0479, COG1053 and COG2009), cytochrome c1 (COG2857), cytochrome c2 (COG3474) and ATP synthase (COG0055, COG0056, COG0355, COG0356 and COG0712). There were also other respiratory enzymes driving oxidative phosphorylation, including lactate dehydrogenase (COG0039 and COG1052), nitrate reductase (COG2181 and COG3005) and glycerol-3-phosphate dehydrogenase (COG0240 and COG0578).

In line with the enrichment of genes associated with oxidative phosphorylation, COGs related to glycolysis were also more abundant in HE (Supplementary file: Supplementary Table 5) including 3-phosphoglycerate kinase (COG0126), 6-phosphofructokinase (COG0205), fructose 1,6-bisphosphate aldolase (COG1830), enolase (COG0148), glucokinase (COG0837), phosphoglycerate mutase (COG0588), pyruvate kinase (COG0469) and triosephosphate isomerase (COG0149).

## Discussion

In this study, we found that diet and exercise interact to alter metabolic markers, inflammatory profiles, and gut microbial composition in mice. Diet played a more important role than exercise in shaping the gut microbiota. We also demonstrated that the beneficial effects of normal diet and exercise can be transmitted via FMT to improve metabolism and inflammatory status in obesity. Finally, we identified several specific microbes associated with this transmissible beneficial effect.

The antibiotics regimen for FMT recipient mice increased their susceptibility to obesity phenotype (Supplementary file: Supplementary Fig. 1). As H_FNE and H_FHE groups exhibited significant reductions in body and fat weight, and improvements in metabolic profiles compared with H group, the beneficial effects of FMT from exercised donors exceedingly counteracted the predisposition to obesity caused by HFD and antibiotics. Therefore, the beneficial effects are potentially even greater than if antibiotics were not used. Last but not least, the larger reduction in food efficacy of H_FNE than H_FHE (Fig. 2B) suggested that transplanted NE microbiota contributed more to the beneficial effects than transplanted HE microbiota.

In terms of diet, ingested food directly interacts with gut microbes and serves as their cultivation medium. Exercise elicits varied physiological responses based on duration and intensity, leading to less predictable microbial fluctuations. These substantial differences in the effects of diet and exercise on the gut microbial community potentially explain our observations that diet is more influential than exercise in altering the gut microbiota.

Bacterial components are known to alter host metabolism and to induce chronic inflammation that underlies obesity. Bacterial lipopolysaccharide^9,11^ and peptidoglycan^32^ represent two classic examples accounting for obesity-related insulin resistance during metabolic endotoxemia, by activating Toll-like receptor 4 (TLR4) and nucleotide-binding oligomerization domain-containing (NOD) proteins (NOD1^33–35^ and NOD2^36,37^), respectively. Therefore, a supplementary experiment probing the expression level of *Tlr4*, *Nod1* and *Nod2* was performed to see the associations between metabolic endotoxemia and FMT (Supplementary file: Supplementary Note 2). Although it was with marginal significance, the lower gene expressions of *Tlr4* (*p* = 0.138, *t*-test vs. H), *Nod1* (*p* = 0.104, *t*-test vs. H) and *Nod2* (*p* = 0.125, *t*-test vs. H) in HE compared to H suggested that exercise apparently reduces metabolic endotoxemia (Supplementary file: Supplementary Fig. 6 and Supplementary Table 6). This result agreed with previous findings in Korean women^38^. Among FMT recipients, the reduced expressions of *Tlr4* and *Nod2* compared to H reflected lower inflammation (Supplementary file: Supplementary Fig. 6), implying that their improved metabolism might associate with the mitigated endotoxemia by receiving FMT from exercised donors. The unaffected expression of *Nod1* requires further exploration.

In agreement with the results of previous studies in murine models^39,40^, the *Akkermansia* and *Lactobacillus* genera were more abundant in exercised mice fed NFD. The increased relative abundance of SMB53 in H rather than N group was consistent with its positive correlation with host adiposity in rats^41^. In addition, the observed increases in *Turicibacter* and *Prevotella* proportion in N compared with H mice were in line with their depletion in HFD-fed mice^42^. With respect to changes in taxa by FMT, the overlap among genera of significant contrasting abundance in FMT recipients (i.e., H_FHE, H_FNE and N_FNE) demonstrated that *Helicobacter*, *Odoribacter* and AF12 are highly associated with FMT from exercised donors. AF12 exhibited about a 100-fold increase in HFD-fed recipients (Supplementary file: Supplementary Table 7). *Helicobacter* and *Odoribacter* demonstrated 2–3 orders of magnitude higher abundance in HFD-fed recipients and an order of magnitude higher abundance in NFD-fed recipients, showing that the diet consumed by FMT recipients plays a role in xenobiotic colonization.

*Helicobacter*, mostly *Helicobacter typhlonius* MU96-1 (Supplementary file: Supplementary Fig. 7A), prevailed among comparisons, reflecting its vulnerability to diet, exercise and even FMT. The co-modulation of *Akkermansia muciniphila* and *H. typhlonius* in intestinal tumor growth has recently been reported^43^. Although a prevalent intestinal colonizer in mice^44^, the role of *H. typhlonius* in the human gut and metabolism is still elusive. The overabundance of *Odoribacter* in exercised mice, in comparison to non-exercised mice (i.e., H-vs-HE and N-vs-NE), highlights the intimate association of *Odoribacter* with exercise. In addition, all FMT recipients (from exercised donors) exhibited significant increase in *Odoribacter* in comparison with controls (i.e., H-vs-H_FHE, H-vs-H_FNE and N-vs-N_FNE), indicating that the increase in *Odoribacter* is induced by FMT regardless of type of diet. As *Odoribacter* is a known producer of short chain fatty acids, such as acetate, propionate, and butyrate^45,46^, increased *Odoribacter* may partly contribute to decreased inflammation, as evidenced by the diminished gene expressions of inflammatory cytokines in mice that were exercised or received FMT from exercised donors. The closest matches for *Odoribacter* OTUs were *Odoribacter* sp. strain Marseille-P2698 and *Odoribacter splanchnicus* strain DJF_B089 (Supplementary file: Supplementary Fig. 7B), both of which have scarcely been investigated in the context of weight control and obesity.

AF12, of the family Rikenellaceae, showed constant overabundance in N, HE, H_FHE and H_FNE groups, when compared with H group, implying a role in weight control. Searching against the Rikenellaceae 16S rRNA gene sequences from NCBI, 62.6% of AF12 OTUs expectedly matched uncultured Rikenellaceae and 28.5% the genus *Alistipes* (Supplementary file: Supplementary Fig. 7C). *Alistipes* has been shown to be more abundant in healthy subjects than in HIV patients^47^, with reduced prevalence in diabetic patients after treatment^48^. Notably, a recent study of a 52-week weight-loss program revealed that a higher baseline proportion of *Alistipes* correlates with better weight loss and maintenance^49^. Similar to *Odoribacter*, Rikenellaceae bacteria are capable of producing butyrate^46^. These observations underlie the beneficial role of AF12.

On functional enrichment analysis, genes associated with electron transport chain (in oxidative phosphorylation) and glycolysis were enriched in HE mice when compared with H mice. Although the mechanism explaining how exercise selects (or alters) gut microbiota remains unclear, our results imply that exercise could establish a microbial community with proficiency in energy production via glycolysis and oxidative phosphorylation, which may contribute to some of the transmissible benefits (e.g., higher carbohydrate catabolism rate as reflected by reduced fat mass and food efficacy) from exercised FMT donors.

There are some limitations to the present study. First, fecal samples contained biological components in addition to bacteria, such as phages, fungi and metabolites, which might account for some of the transmissible benefits via FMT. The application of metagenomics to this study allowed for the dissection of the contribution of each fecal component to the beneficial effect and the potential involvement of bacteria was revealed. Second, to maximize the success rate of transplantation, daily FMT (5 days a week) was performed, which may not be feasible in clinical practice, although this schedule has been utilized in recent clinical trials in inflammatory bowel disease^50^. A standard protocol for workable FMT frequency with guaranteed successful transplantation warrants further investigation. Third, our study was entirely based on murine models and extrapolation to humans must be performed with caution, although the proof of concept approach warrants appropriate human studies. Fourth, the running velocity, 18 m/min, represents the critical speed for the C57BL/6J mouse^51^, which is the maximum velocity a muscle group can maintain without exhaustion^52^. Therefore, the exercise regimen in our experimental setting (i.e., running at critical speed for 30 mins per day, 5 days per week) is very likely challenging for the ordinary human population. A systematic evaluation of exercise types, intensity, duration and practicality would help standardize the criteria for selecting desirable and effective FMT donors. Lastly, as the identified *Helicobacter*, *Odoribacter* and AF12 species are either recalcitrant to cultivation (strictly anaerobic) or without available isolates, we could not explore these further. However, the results are encouraging and merit further investigation.

The present study demonstrates for the first time that FMT from donors with normal diet and exercise confers metabolic benefits to HFD-fed recipients. Although diet played a more important role in shaping the gut microbiota in our study compared to exercise, the combined effect of healthy diet and exercise may constitute to an ideal gut microbiota for facilitating weight control. In conclusion, although this study is based on murine model and clinical effects in human trials remain to be verified, our findings provide proof of concept that obesity in a mammalian host can respond to FMT from donors with balanced diet, regular exercise or, even better, a combination of both.

## Methods

### Study design

In this study, mice were divided into seven groups (*n* = 47), including H (high fat diet, *n* = 6), HE (H with exercise, *n* = 7), N (normal fat diet, *n* = 7), NE (N with exercise, *n* = 6), H_FHE (H receiving FMT from HE, *n* = 7), H_FNE (H receiving FMT from NE, *n* = 7) and N_FNE (N receiving FMT from NE, *n* = 7). H represents the diet-induced obesity group independent from external treatment (e.g., exercise or FMT). N represents the treatment-naïve group with normal fat diet. Comparisons were performed for each group versus H to monitor the differential outcomes of metabolic and inflammatory profiles and gut microbiota under given treatments. Mice were randomly assigned to each group except for those in the exercised groups identified during 2-week assimilation training. Details of study design are shown in Fig. 1. Animal management, exercise regimen and FMT protocol are described below.

### Animal model

All experimental procedures were approved by the Institutional Animal Care and Utilization Committee of Taichung Veterans General Hospital (La-1031216 and La-1041342) and performed in accordance with institutional guidelines. Male C57BL/6JNarl mice (*n* = 49), aged five weeks, were received from the National Laboratory Animal Center (Taipei, Taiwan). These mice were initially group housed in 7 cages (7 mice per cage) with free access to water and food. The mice in 4 cages were provided high fat diet (HFD; composition 60 kcal% fat, 20 kcal% carbohydrates, 20 kcal% protein; 5.24 kcal/g total energy content; Research Diets Inc., New Brunswick, NJ, USA) and the mice in 3 cages were provided normal fat diet (NFD; composition 11 kcal% fat, 63 kcal% carbohydrates, 26 kcal% protein; 3.25 kcal/g total energy content; Fwusow Industry, Taichung, Taiwan). The housing conditions included constant temperature of 22 ± 1 °C and regular 12-hour light/dark cycle (lights on 8 PM to 8 AM).

After a one-week acclimation period, all mice underwent assimilation training for 2 weeks to identify those competent to be enrolled in the exercised groups. Assimilation training was carried out individually for 39 minutes every Monday and Tuesday on a motorized treadmill outside the cage, starting with a 5-minute warm-up at 7 m/min that was followed by acceleration at 3 m/min every minute to a maximum speed of 18 m/min. After 2-week assimilation training, the mice were reallocated into 7 groups (H, HE, H_FHE, H_FNE, N, NE and N_FNE). Fourteen mice adaptable to treadmill running were assigned to exercised groups (7 in HE and 7 in NE) and underwent formal exercise regimen (18 m/min, 30 min/day, 5 days/week)^53^ following the same warm-up program as during assimilation training for 16 weeks. During exercise sessions, HE and NE mice were pulled from their respective cages and exercised on a motorized 5-lane treadmill at zero-percent incline. The chosen exercise paradigm aimed to create a high impact training model for C57BL/6J mice^51,54^. Forty-nine mice were initially enrolled in the experiment. Two were removed from downstream analysis, of which one in the H group was recalcitrant to HFD (increased 8 g by the end of experiment) and one in the NE group died during exercise at week 15.

### Fecal sample collection and FMT

For comparative purposes, mice feces were collected twice for 16S ribosomal RNA (rRNA) gene amplicon sequencing, once at the beginning of week 12 (i.e., before FMT) and once at the end of week 24 (i.e., after FMT). About 100 mg of feces from each mouse were stored in 1 mL InhibitEX Buffer (Qiagen, Gaithersburg, MD, USA) at −80 °C for 1 week or less until DNA extraction.

After four weeks of formal exercise regimen (i.e., at week 12), feces from exercised mice were collected fresh for daily FMT, which was conducted under sterile conditions under laminar flow hood. Feces from exercised mice were pooled by cage, and 100 mg (about 5–6 fecal pellets) were re-suspended in 1 mL sterile saline. The solution was vigorously mixed for 10 seconds before centrifugation at 800 × g for 3 min. The supernatant (about 500–600 μL) was collected and administered by oral gavage within 10 min to minimize changes in microbial contents^55^.

Among the sedentary mice, 14 HFD-fed and 7 NFD-fed mice were randomly designated as FMT recipients (7 H_FHE, 7 H_FNE and 7 N_FNE) (Fig. 1). According to the established microbial depletion and recolonization protocol^56–59^, a combination of ciprofloxacin (0.2 g/L) and metronidazole (1 g/L) was added to the drinking water of FMT recipients for two days (i.e., the weekend of week 11) before FMT, to ensure that the outcomes are compatible with clinical guidelines for FMT in humans^27^. Ciprofloxacin plus metronidazole was chosen because it is one of the first-line antibiotic regimens recommended for the treatment of abdominal infections in adults^60^. Non-FMT recipients did not receive any antibiotics. Antibiotics were obtained from Sigma-Aldrich Corp. (St. Louis, MO, USA). From week 12, FMT was conducted each weekday, with each recipient administered 100 μL fecal supernatant by oral gavage until week 24.

### Metabolic marker profiles of mice

For each mouse, body weight was measured weekly and intraperitoneal glucose tolerance test (IPGTT) was conducted monthly. For IPGTT, mice received intraperitoneal injection of 20% glucose with normal saline (2 g glucose/kg body mass) after 16-hour fast, and blood glucose levels at 0, 30, 60, 90, and 120 minutes were measured using GM700 (BIONIME, Taichung, Taiwan).

Food consumption per group was recorded weekly, to monitor their feeding behavior throughout the FMT experimental period. Food efficacy was estimated for each group as grams of body weight gain per 100 g food consumed.

At the end of the experimental period, mice were euthanized by carbon dioxide inhalation after overnight fast. The chest was opened and blood was drawn from the left ventricle apex of heart. Serum was obtained by low-speed centrifugation (3000 × g, 10 min, 4 °C), then stored at −80 °C and delivered to the National Laboratory Animal Center within one week for measurement of fasting blood glucose, serum alanine aminotransferase (ALT) activity, and low-density lipoprotein (LDL) cholesterol determinations using a Hitachi 7080 automatic chemistry analyzer (Hitachi Co., Ltd., Tokyo, Japan). Intra-abdominal fat and epididymal fat were manually separated and collectively weighed as fat weight.

### qPCRs of inflammatory cytokines in mice

Chronic inflammation in lipid is associated with HFD-induced obesity^61^. To quantitatively compare inflammation status of mice, the gene expression of related inflammatory cytokines, including tumor necrosis factor (*Tnf*), interleukin 1 alpha (*Il1a*) and peroxisome proliferator-activated receptor gamma (*Pparg*)^62,63^, were measured in liver by quantitative polymerase chain reactions (qPCRs).

Total RNA was extracted from liver tissues and kept in TriPure isolation reagent (Roche Molecular Biochemicals, Mannheim, Germany). Of the total RNA, 1 μg was treated with 10 units of RQ1 RNase-Free DNase (Promega Corp., Madison, WI, USA) and purified with phenol-chloroform. The purified RNA was reverse transcribed into cDNA using Advantage RT-for-PCR Kit (Clontech, Palo Alto, CA, USA). FastStart Universal Probe Master and Universal Probe Library probes (Roche Molecular Biochemicals) were used in qPCRs. For each inflammatory cytokine gene (i.e., *Tnf*, *Il1a* and *Pparg*), qPCRs were performed in triplicate for each of the 47 cDNA pools along with a no template control in parallel. The primers and probes are listed in Supplementary Table 8 (Supplementary file). The qPCR cycling conditions were: 50 °C for 2 min, 95 °C for 10 min and 40 cycles at 95 °C for 15 sec, with a final extension at 60 °C for 1 min using a StepOnePlus Real-Time PCR System (Applied Biosystems, Foster City, CA, USA). Relative gene expression was quantified by the comparative *C*_T_ (ΔΔ*C*_T_) method, employing 18S rRNA gene (*Rn18s*) as internal control for normalization.

### Statistical analysis of mice metabolic parameters and gene expression data

Differences in body weights among groups were evaluated by two-way ANOVA. For the remaining metabolic parameters and gene expression data (including fat weight, fasting blood glucose, IPGTT, *Tnf* expression, *Il1a* expression, *Pparg* expression, ALT and LDL), to avoid potential bias from sporadic outliers on statistical tests (e.g., one *Tnf* expression was higher than average by 99 folds), the 5% truncated mean (data within 5–95% quantile) was applied. The truncated mean is a robust estimator of the arithmetic mean because it is more resistant to outliers while providing a reasonable estimate of the central tendency of data^64^. The 50% truncated mean is equivalent to the median, and therefore the truncated mean is often regarded as a compromise between the arithmetic mean and median. With H the diet-induced obesity group, Student’s *t*-test was used to assess the respective difference in the means of other groups versus H, to understand the outcomes of external treatments designed to deal with obesity.

### Histological examination

HFD is associated with fatty liver in mouse and humans^29,30^. Therefore, mice liver and fat pad tissues were subjected to histological examination to confirm the effectiveness of HFD in terms of adipose accumulation. After euthanasia, mice livers and fat pad tissues were collected in 10% formaldehyde for histological examination by Bestjet Biotechnology Co., Ltd. (Taipei, Taiwan). Haematoxylin and Eosin (H&E) staining was applied to both tissue types and oil red O staining was applied to fat pad tissues. After staining, tissues were examined under light microscope at 200× magnification.

### Fecal DNA extraction

Fecal DNA was extracted using QIAamp Fast DNA Stool Mini Kit (Qiagen). DNA quality and quantity were determined by agarose gel electrophoresis and NanoDrop ND-1000 (Thermo Scientific, Wilmington, DE, USA). DNA was stored at −80 °C until 16S rRNA gene amplicon sequencing (at least 500 ng per sample).

### 16S rRNA gene amplicon sequencing and analysis

The hypervariable regions V3–V4 of bacterial 16S rRNA genes were amplified by PCR using bar-coded universal primers 341F (F, forward primer; 5′-CCTACgggNggCWgCAg-3′) and 805R (R, reverse primer; 5′-gACTACHVgggTATCTAATCC-3′). Library construction and sequencing of amplicon DNA samples were done by Genomics BioScience (Taipei, Taiwan). A pair-end library (insert size of 465 bp for each sample) was constructed using MiSeq Reagent Kit v3 according to the manufacturer’s instructions (Illumina, Wilmington, DE, USA) and high-throughput sequencing was performed on Illumina MiSeq2000 platform (Illumina).

Bioinformatics analysis of 16S rRNA gene amplicons was performed by Germark Biotechnology (Taichung, Taiwan). Briefly, USEARCH (v7.0.1090) was used to merge pair-end reads on a per-sample basis, setting 8 bp as the minimum overlap of read pair. Mothur (v1.34.3) was used for quality-filtering to retain reads with: (1) a length of 400–550 bp, (2) a minimum average quality score of 27, (3) no ambiguous base and (4) homopolymers not exceeding 8 bp. Chimera detection was performed by UCHIME^65^ as implemented in USEARCH using reference mode and 3% minimum divergence. Quality-filtered and non-chimeric reads were further analyzed according to UPARSE pipeline^66^ to generate OTUs per sample (97% identity level). OTU representative sequences were searched against the Greengenes 13_5 database using USEARCH global alignment to identify the corresponding taxonomy of the best hit. Any OTU without a hit or with only a weak hit (i.e., the average of % sequence identity and % alignment coverage <93) was excluded from following analyses.

### Statistical and bioinformatics analyses of microbiota

All statistical analyses were performed using R (https://www.r-project.org/), unless otherwise specified. PCoA was conducted with R package phyloseq based on OTU-level Bray–Curtis distance measure. Between-group inertia percentage was evaluated using R package ade4 with Monte-Carlo test (with 1000 permutations) to determine the *p* value of PCoA results. Two-way PERMANOVA was performed by R package vegan using OTU-level Bray–Curtis distance matrix with 999 permutations. Shannon diversity index was estimated at OTU level by R package phyloseq and tested for associations with sampling time (before and after FMT), diet, exercise and FMT status by linear modeling, in which the data of H_FHE and H_FNE before FMT were categorized into H group and that of N_FNE before FMT into N group. To identify genera differentially enriched in the between-group comparisons, LEfSe was applied with α = 0.05 (Kruskal–Wallis and Wilcoxon tests) and effect size threshold of two on LDA using the stand-alone implementation (https://bitbucket.org/nsegata/lefse). Enriched genera identified by LEfSe were further filtered by a group-mean difference in relative abundance larger than 10^−5^ to exclude genera absent in one group and minutely abundant in another. To deliver a more intuitive perception of contrasting abundance, LEfSe results were visualized by plotting the log-2 ratio of average relative abundance between groups rather than the LDA score. The abundance profile of functional genes (i.e., COGs) of each microbial community was predicted using PICRUSt (v1.0.0). To identify functional genes with differential abundances, enrichment analysis was performed by a two-group comparison for medians using two-tailed Wilcoxon test with a Benjamini-Hochberg false discovery rate correction to adjust q-values for multiple testing. The R code for all statistical analyses is available in Supplementary dataset, and was created by the R package knitr^67^.

## Electronic supplementary material

Supplementary Information

Supplementary Dataset

## Data Availability

Sequence files and metadata of all samples have been deposited in National Center for Biotechnology Information Sequence Read Archive under accession number SRP102269, affiliated with BioProject PRJNA379878. The metadata, OTU and taxonomy tables, and COGs and annotation tables (predicted by PICRUSt) have also been deposited in Figshare (https://doi.org/10.6084/m9.figshare.5513548), and serve as input data for the R code in Supplementary dataset used to perform all statistical analyses in this manuscript.
