# Supplementary material for: Fecal microbiota transplantation confers beneficial metabolic effects of diet and exercise on diet-induced obese mice
Source: Sci Rep. 2018 Oct 23;8:15625. doi: 10.1038/s41598-018-33893-y (PMC6199268; doi:10.1038/s41598-018-33893-y)
Supplement: Supplementary file 1 — Supplementary Information [file 41598_2018_33893_MOESM1_ESM.pdf]

Supplementary file – Supplementary information

Fecal microbiota transplantation confers beneficial metabolic effects of diet and exercise on diet-induced obese mice

Zi-Lun Lai[1†], Ching-Hung Tseng[2†], Hsiu J. Ho[1], Cynthia K. Y. Cheung[3], Jian-Yong Lin[2], Yi-Ju Chen[4,5], Fu-Chou Cheng[6], Yao-Chun Hsu[7,8], Jaw-Town Lin[9,10], Emad M. El-Omar[11*] and Chun-Ying Wu[1,4,8,12-15*]

[1]Division of Gastroenterology, Taichung Veterans General Hospital, [2]Germark Biotechnology Co., Ltd., Taichung, Taiwan; [3]Institute of Digestive Disease, the Chinese University of Hong Kong, Shatin, Hong Kong; [4]Faculty of Medicine and Graduate Institute of Clinical Medicine, National Yang-Ming University, Taipei, Taiwan; [5]Department of Dermatology, [6]Stem Cell Center, Department of Medical Research, Taichung Veterans General Hospital, Taichung, Taiwan; [7]Department of Internal Medicine, E-Da Hospital/I-Shou University, Kaohsiung, Taiwan; [8]Graduate Institute of Clinical Medicine, China Medical University, Taichung, Taiwan; [9]School of Medicine, Fu Jen Catholic University, New Taipei City, Taiwan; [10]Institute of Population Health Sciences, National Health Research Institutes, Miaoli, Taiwan; [11]Microbiome Research Centre, St George and Sutherland Clinical School, University of New South Wales, Sydney, Australia; [12]Division of Translational Research, Taipei Veterans General Hospital; [13]National Institute of Cancer Research, National Health Research Institutes, Miaoli, Taiwan; [14]Department of Public Health, China Medical University, [15]Department of Life Sciences and Rong Hsing Research Center for Translational Medicine, National Chung-Hsing University, Taichung, Taiwan.

[†]Authors contribute equality to this work.

[*]Authors share co-corresponding authorship.

Note

Supplementary Note 1. Effects of two-day antibiotics exposure on obesity.
Supplementary Note 2. qPCR of *Tlr4*, *Nod1* and *Nod2*.

Figure

Supplementary Figure 1. Body weight gain and IPGTT results in H and H_ABX.
Supplementary Figure 2. IPGTT curves for each mouse group.
Supplementary Figure 3. Histological examination of liver and fat pad tissues.
Supplementary Figure 4. Interaction plot of diet and exercise to Shannon diversity.
Supplementary Figure 5. LEfSe comparisons among FMT donors and recipients.
Supplementary Figure 6. Expression of *Tlr4*, *Nod1* and *Nod2*.
Supplementary Figure 7. Detailed strain information of OTUs in NCBI database.

Table

Supplementary Table 1. Body weights data of H and H_ABX.
Supplementary Table 2. IPGTT results of H and H_ABX.
Supplementary Table 3. Mouse physiological parameters and gene expression data.
Supplementary Table 4. COGs of energy production enriched in HE than H.
Supplementary Table 5. COGs of carbohydrate metabolism enriched in HE than H.
Supplementary Table 6. Expression data of *Tlr4*, *Nod1* and *Nod2*.
Supplementary Table 7. Magnitude of abundance changes in taxa by FMT.
Supplementary Table 8. List of primers and probes used in qPCR.

References

Note

Supplementary Note 1. Effects of two-day antibiotics exposure on obesity.

Antibiotics ciprofloxacin (0.2 g/L) and metronidazole (1 g/L) was added to the drinking water of FMT recipients for two days before FMT was performed to ensure that the outcomes are compatible with the clinical guidelines for FMT in humans.

Early-life antibiotics exposure predisposes hosts to obesity[1]. To verify this effect, a supplementary H_ABX group of mice (n = 8) was raised in identical conditions to H but underwent the same antibiotics regimen as FMT recipients and received daily gavage of normal saline (100 μl) as vehicle FMT. H_ABX mice were raised for 8 weeks as they were assumed to have no differences to H regarding phenotypic and metabolic profiles. H_ABX mice were exposed to antibiotics for two days prior to week 6 as they did not receive assimilation training.

Throughout the feeding period, the food consumption (adjusted to n = 8) of H mice was 144 g/week, and that of H_ABX mice was 136 g/week; the food efficacy of H mice was 6.045 (gw weight gain per 100 g food consumed) and that of H_ABX was 6.072. The results showed no difference in feeding behaviors of H and H_ABX.

H and H_ABX mice after 8-week high-fat diet were compared for body weight gains and IPGTT results (Supplementary Table 1 and 2). As the results, differences in body weight gain were not significant (Supplementary Figure 1A), but that in glucose tolerance were significantly higher in H_ABX than H (Supplementary Figure 1B and 1C). The results were in agreement with the previous study[2], and suggested that the applied antibiotics regimen predisposed high fat diet-fed mice to physiological changes associated with obesity.

Supplementary Note 2. qPCR of *Tlr4*, *Nod1* and *Nod2*.

Bacterial lipopolysaccharide[3] and peptidoglycan[4] are known to alter host metabolism and inflammation during metabolic endotoxemia. Bacterial lipopolysaccharide triggers downstream pathways by activating Toll-like receptor 4 (TLR4) and peptidoglycan by activating nucleotide-binding oligomerization domain-containing (NOD) proteins, NOD1 and NOD2. To evaluate the associations between FMT and metabolic endotoxemia, the gene expressions of *Tlr4*, *Nod1* and *Nod2* were determined using quantitative polymerase chain reaction (qPCR) in mice liver tissue.

The qPCR protocols for *Tlr4*, *Nod1* and *Nod2* were identical to those mentioned in main text, but glyceraldehyde 3-phosphate dehydrogenase (*Gapdh*) was used as the internal control. Three mice from H_FHE and H_FNE (i.e., the major treatment groups) and two mice from other groups were randomly selected. Expression data (expressed in $2^{-\Delta\Delta Ct}$) are in Supplementary Table 6 and the qPCR primers for *Tlr4*, *Nod1*, *Nod2* and *Gapdh* are in Supplementary Table 8.

Figure

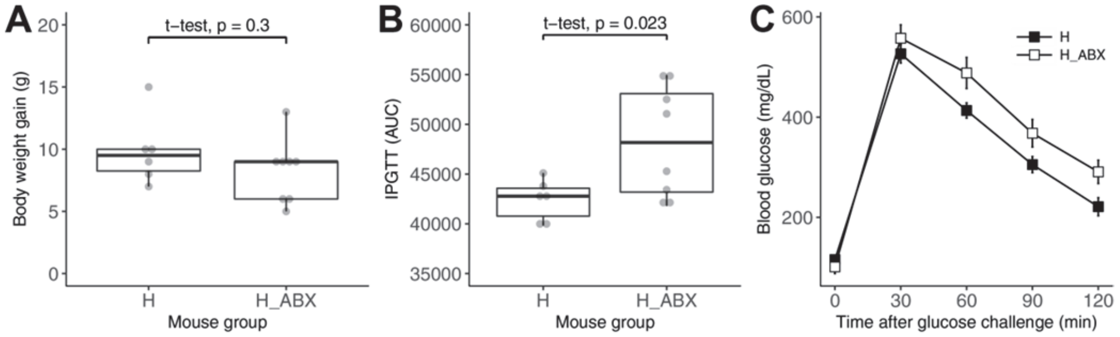

Supplementary Figure 1. Body weight gain and IPGTT results in H and H_ABX. Body weight gains between H (n = 6) and the additional H_ABX group (n = 8) from week 8 to 14 (see Supplementary Note 1) was tested by Student's *t*-test. Data points for body weight gain and AUC after 8-week high-fat diet are labeled with semi-transparency. Time-course data of IPGTT are mean ± SE every 30 minutes until 2 hours after glucose challenge.

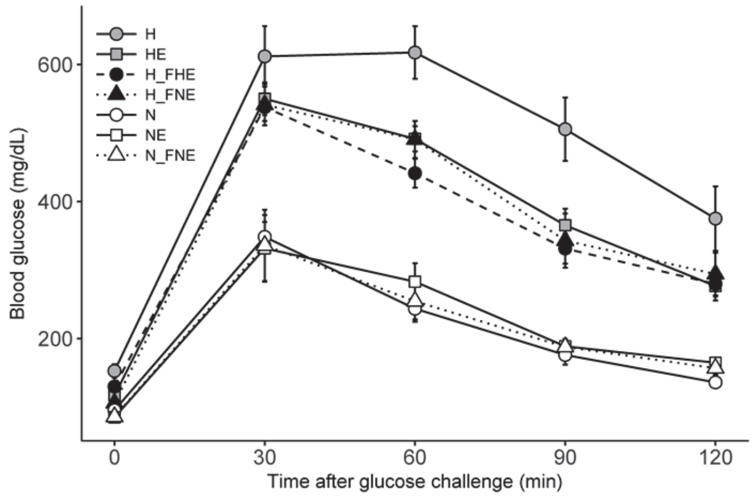

Supplementary Figure 2. IPGTT curves for each mouse group. IPGTT was measured monthly throughout the experiment, and the last measurement (at week 24) was presented. The data are mean ± SE, measured every 30 minutes until 2 hours after glucose challenge.

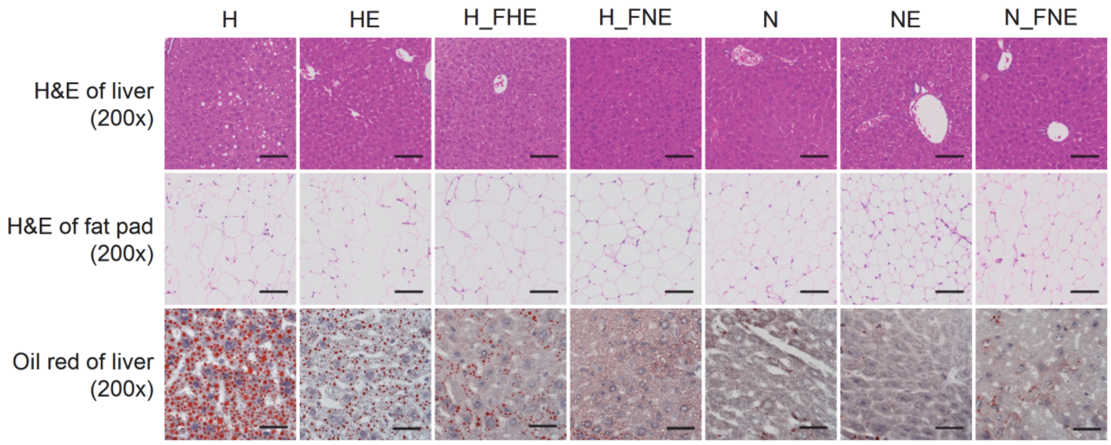

Supplementary Figure 3. Histological examination of liver and fat pad tissues. Haematoxylin & eosin (H&E) stainning were applied to liver and fat pad tissues. Oil red O staining was applied to fat pad tissue as well. Mouse groups are labeled on top of each column in figure. Scale bar represents 100 µm.

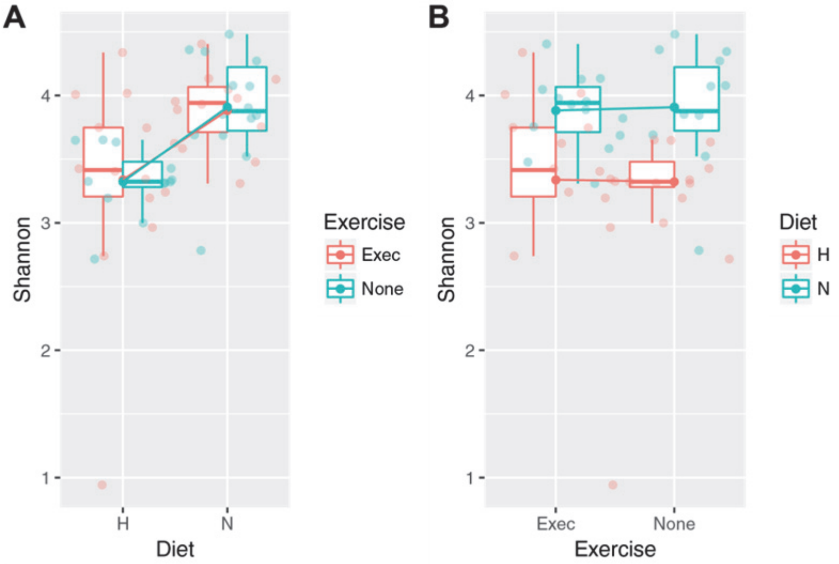

Supplementary Figure 4. Interaction plot of diet and exercise to Shannon diversity. (A) Different diet types were compared with exercise status. (B) Different exercise status were compared with diet types.

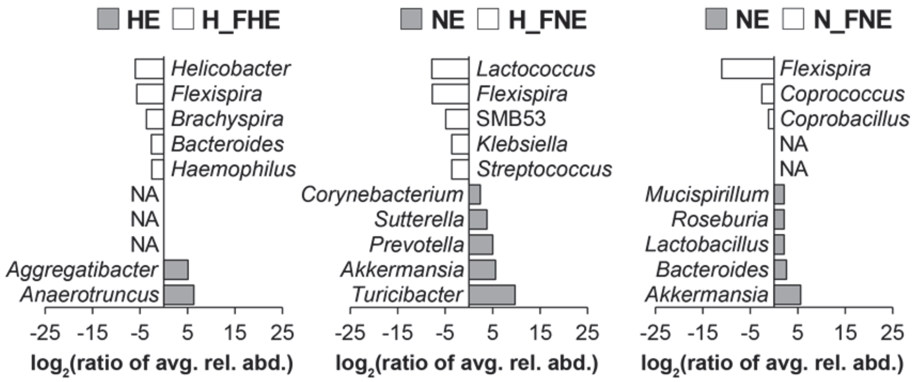

Supplementary Figure 5. LEfSe comparisons among FMT donors and recipients. Top five genera with contrasting abundances bewteen compared groups are listed. NA, not applicable.

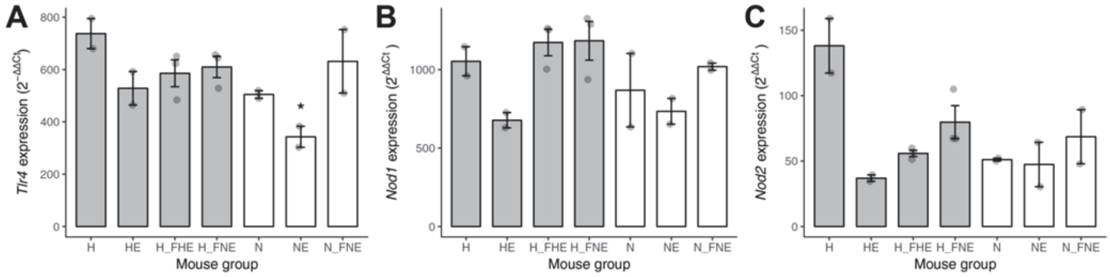

Supplementary Figure 6. Expression of *Tlr4*, *Nod1* and *Nod2*. (A) Expression of *Tlr4* gene. (B) Expression of *Nod1* gene. (C) Expression of *Nod2* gene. Data points are labeled with semi-transparency. The data are mean ± SE. *$p < 0.05$, **$p < 0.01$, ***$p < 0.001$ (Student's *t*-test, vs. H).

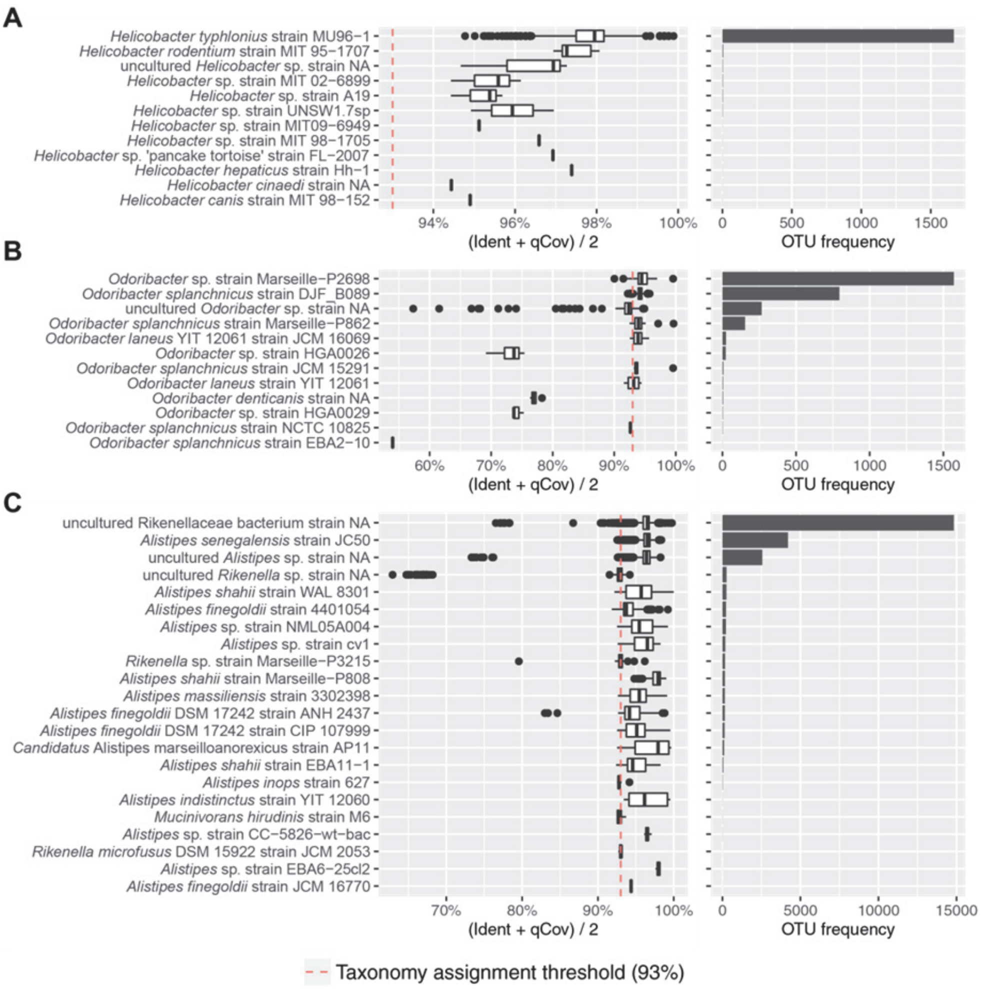

Supplementary Figure 7. Detailed strain information of OTUs in NCBI database. (A) *Helicobacter* OTUs. (B) *Odoribacter* OTUs. (C) AF12 OTUs. The representative sequences of OTUs were search against NCBI RefSeq database using BLASTn. The distribution of matched identity (Ident) and query coverage (qCov) and respective frequency of matched taxa (in strain, if available) were summarized.

Table

Supplementary Table 1. Body weight of H and H_ABX. Data are mouse weekly body weight (g) of H and H_ABX. H received no antibiotics exposure, and H_ABX was exposed to ciprofloxacin (0.2 g/L) and metronidazole (1 g/L) through drinking water for two days before week 6.

Group	Mouse ID	Week										
		6	7	8	9	10	11	12	13	14	15	16
H	H-0401	Assimilation training		21	24	23	25	26	28	28	31	31
	H-0402			23	26	25	25	27	28	29	31	30
	H-0403			20	23	22	22	23	24	25	28	28
	H-0404			23	28	28	29	32	33	36	38	38
	H-0405			20	23	23	24	26	26	28	30	30
	H-0407			21	24	23	24	26	27	28	29	30
	H-0406	Mouse recalcitrant to HFD										
H_ABX	HFD 0101	24	24	24	23	25	26	28	28	30	Sacrificed	
	HFD 0102	19	19	19	22	24	25	26	26	28		
	HFD 0103	20	21	18	20	22	23	25	23	26		
	HFD 0104	22	23	23	24	27	28	29	30	31		
	HFD 0105	21	23	22	24	25	26	28	28	30		
	HFD 0106	21	23	22	26	26	28	29	29	30		
	HFD 0107	21	17	18	27	30	31	31	32	34		
	HFD 0108	21	18	20	21	22	26	26	25	26		

Supplementary Table 2. IPGTT results of H and H_ABX. Time course data of blood glucose concentration (mg/dL) and AUC of H and H_ABX are presented.

Group	Mouse ID	Time after glucose challenge (min)					AUC
		0	30	60	90	120	
H (week 16)	H-0401	110	568	410	350	241	45105
	H-0402	108	526	441	298	222	42900
	H-0403	100	591	348	269	161	40155
	H-0404	135	503	454	343	185	43800
	H-0405	125	494	402	253	232	39825
	H-0407	117	476	425	319	287	42660
	H-0406	Mouse recalcitrant to HFD					NA
H_ABX (week 14)	HFD 0101	90	539	461	331	267	45285
	HFD 0102	112	500	372	351	276	42510
	HFD 0103	14	516	513	262	189	41775
	HFD 0104	128	654	425	385	348	51060
	HFD 0105	127	485	538	534	412	54795
	HFD 0106	121	493	399	358	274	43425
	HFD 0107	98	598	585	387	263	52515
	HFD 0108	121	675	612	335	298	54945

Supplementary Table 3. Mouse physiological parameters and gene expression data. Data are 5% truncated mean ± SE, with truncated sample size *n* in parenthesis.

Group	Fat weight (g)	Fasting blood glucose (mg/dL)	IPGTT (AUC)	*Tnf* ($2^{-\Delta\Delta Ct}$)
H	2.00 ± 0.13 (4)	155.8 ± 5.0 (4)	59486.3 ± 2627.4 (4)	0.17 ± 0.04 (4)
HE	0.95 ± 0.05 (5)	123.4 ± 4.4 (5)	48993.0 ± 899.1 (5)	0.25 ± 0.05 (5)
H_FHE	1.30 ± 0.16 (5)	127.0 ± 16.2 (5)	44547.5 ± 1976.4 (6)	0.06 ± 0.01 (5)
H_FNE	1.08 ± 0.25 (5)	103.0 ± 9.7 (5)	47178.0 ± 2568.5 (5)	0.10 ± 0.02 (5)
N	0.33 ± 0.03 (5)	92.4 ± 6.6 (5)	25758.0 ± 1131.7 (5)	0.12 ± 0.02 (5)
NE	0.36 ± 0.02 (5)	85.3 ± 1.8 (4)	28567.5 ± 1626.5 (4)	0.19 ± 0.07 (4)
N_FNE	0.26 ± 0.03 (5)	86.2 ± 1.5 (5)	25848.0 ± 823.8 (5)	0.12 ± 0.01 (5)
**Group**	***Il1a*** **($2^{-\Delta\Delta Ct}$)**	***Pparg*** **($2^{-\Delta\Delta Ct}$)**	**ALT (U/L)**	**LDL (mg/dL)**
H	0.27 ± 0.02 (4)	0.17 ± 0.02 (4)	40.70 ± 2.68 (4)	22.65 ± 0.48 (4)
HE	0.18 ± 0.02 (5)	0.10 ± 0.01 (5)	25.68 ± 1.21 (5)	15.28 ± 0.97 (5)
H_FHE	0.17 ± 0.01 (5)	0.13 ± 0.01 (5)	22.48 ± 0.69 (5)	14.06 ± 1.05 (5)
H_FNE	0.16 ± 0.01 (5)	0.15 ± 0.02 (5)	23.48 ± 0.32 (5)	14.36 ± 0.18 (5)
N	0.24 ± 0.02 (5)	0.15 ± 0.01 (5)	18.56 ± 0.95 (5)	13.02 ± 0.52 (5)
NE	0.32 ± 0.07 (4)	0.20 ± 0.05 (4)	22.03 ± 1.40 (4)	13.40 ± 0.60 (4)
N_FNE	0.17 ± 0.02 (6)	0.12 ± 0.01 (5)	22.76 ± 1.52 (5)	11.92 ± 0.67 (5)

Supplementary Table 4. COGs of energy production enriched in HE than H. Data were derived from two-tailed Wilcoxon test on group medians with *p* values ajusted by Benjamini-Hochberg (BH) false discovery rate for multiple testing. Difference between group medians (HE minus H) and fold change (HE divide H) were also showned. COGs were sorted in ascending order of adjusted *p* values with lexical order of COG descriptions.

#	COG ID	COG description	Adjusted *p* (BH)	HE minus H	HE divide H
1	COG1271	Cytochrome bd-type quinol oxidase, subunit 1	0.004	1.9E-04	2.9
2	COG1294	Cytochrome bd-type quinol oxidase, subunit 2	0.004	1.7E-04	2.9
3	COG0712	F0F1-type ATP synthase, delta subunit (mitochondrial oligomycin sensitivity protein)	0.004	1.0E-04	1.3
4	COG0247	Fe-S oxidoreductase	0.004	3.0E-04	2.5
5	COG0731	Fe-S oxidoreductases	0.004	1.7E-04	3.0
6	COG1148	Heterodisulfide reductase, subunit A and related polyferredoxins	0.004	1.2E-04	2.7
7	COG2048	Heterodisulfide reductase, subunit B	0.004	9.6E-05	3.1
8	COG1150	Heterodisulfide reductase, subunit C	0.004	1.1E-04	3.0
9	COG1726	Na+-transporting NADH:ubiquinone oxidoreductase, subunit NqrA	0.004	1.7E-04	3.2
10	COG1805	Na+-transporting NADH:ubiquinone oxidoreductase, subunit NqrB	0.004	1.7E-04	3.2
11	COG2869	Na+-transporting NADH:ubiquinone oxidoreductase, subunit NqrC	0.004	1.7E-04	3.2
12	COG1347	Na+-transporting NADH:ubiquinone oxidoreductase, subunit NqrD	0.004	1.7E-04	3.2
13	COG2209	Na+-transporting NADH:ubiquinone oxidoreductase, subunit NqrE	0.004	1.7E-04	3.2
14	COG2871	Na+-transporting NADH:ubiquinone oxidoreductase, subunit NqrF	0.004	1.7E-04	2.9
15	COG3005	Nitrate/TMAO reductases, membrane-bound tetraheme cytochrome c subunit	0.004	9.5E-05	3.7
16	COG1866	Phosphoenolpyruvate carboxykinase (ATP)	0.004	1.3E-04	1.6
17	COG5016	Pyruvate/oxaloacetate carboxyltransferase	0.004	1.9E-04	1.7
18	COG1053	Succinate dehydrogenase/fumarate reductase, flavoprotein subunit	0.004	1.8E-04	1.4
19	COG1062	Zn-dependent alcohol dehydrogenases, class III	0.004	8.7E-07	10.0
20	COG1143	Formate hydrogenlyase subunit 6/NADH:ubiquinone oxidoreductase 23 kD subunit (chain I)	0.004	4.3E-05	1.1
21	COG3474	Cytochrome c2	0.005	7.7E-08	14.5
22	COG4237	Hydrogenase 4 membrane component (E)	0.005	8.0E-05	3.2
23	COG0680	Ni,Fe-hydrogenase maturation factor	0.005	6.0E-05	3.2
24	COG0778	Nitroreductase	0.005	5.6E-04	1.3
25	COG3051	Citrate lyase, alpha subunit	0.005	4.3E-06	3.6
26	COG3052	Citrate lyase, gamma subunit	0.005	4.4E-06	3.7
27	COG1035	Coenzyme F420-reducing hydrogenase, beta subunit	0.005	1.5E-04	2.8
28	COG0056	F0F1-type ATP synthase, alpha subunit	0.005	7.1E-05	1.2
29	COG0055	F0F1-type ATP synthase, beta subunit	0.005	7.1E-05	1.2
30	COG0355	F0F1-type ATP synthase, epsilon subunit (mitochondrial delta subunit)	0.005	7.1E-05	1.2
31	COG0356	F0F1-type ATP synthase, subunit a	0.005	7.1E-05	1.2
32	COG0578	Glycerol-3-phosphate dehydrogenase	0.005	1.6E-04	2.8
33	COG0538	Isocitrate dehydrogenases	0.005	9.8E-05	1.3
34	COG0039	Malate/lactate dehydrogenases	0.005	1.1E-04	1.2
35	COG1252	NADH dehydrogenase, FAD-containing subunit	0.005	1.7E-04	2.8
36	COG0374	Ni,Fe-hydrogenase I large subunit	0.005	7.9E-05	3.1
37	COG1740	Ni,Fe-hydrogenase I small subunit	0.005	7.8E-05	3.1

38	COG0280	Phosphotransacetylase	0.005	2.1E-04	1.4
39	COG0348	Polyferredoxin	0.005	1.7E-04	1.4
40	COG1014	Pyruvate:ferredoxin oxidoreductase and related 2-oxoacid:ferredoxin oxidoreductases, gamma subunit	0.005	4.0E-04	1.8
41	COG1048	Aconitase A	0.006	4.7E-05	1.1
42	COG0240	Glycerol-3-phosphate dehydrogenase	0.006	5.3E-05	1.1
43	COG1969	Ni,Fe-hydrogenase I cytochrome b subunit	0.006	2.6E-05	4.6
44	COG2878	Predicted NADH:ubiquinone oxidoreductase, subunit RnfB	0.006	8.3E-05	1.3
45	COG4660	Predicted NADH:ubiquinone oxidoreductase, subunit RnfE	0.006	7.8E-05	1.2
46	COG3069	C4-dicarboxylate transporter	0.006	1.4E-05	11.5
47	COG1908	Coenzyme F420-reducing hydrogenase, delta subunit	0.006	3.5E-05	2.6
48	COG1052	Lactate dehydrogenase and related dehydrogenases	0.006	1.1E-04	1.1
49	COG3488	Predicted thiol oxidoreductase	0.006	5.9E-05	2.9
50	COG0674	Pyruvate:ferredoxin oxidoreductase and related 2-oxoacid:ferredoxin oxidoreductases, alpha subunit	0.006	3.8E-04	1.5
51	COG1013	Pyruvate:ferredoxin oxidoreductase and related 2-oxoacid:ferredoxin oxidoreductases, beta subunit	0.006	3.4E-04	1.8
52	COG0479	Succinate dehydrogenase/fumarate reductase, Fe-S protein subunit	0.006	1.6E-04	2.3
53	COG0372	Citrate synthase	0.007	3.6E-05	1.1
54	COG2009	Succinate dehydrogenase/fumarate reductase, cytochrome b subunit	0.007	1.9E-06	3.5
55	COG1951	Tartrate dehydratase alpha subunit/Fumarate hydratase class I, N-terminal domain	0.007	8.0E-05	1.2
56	COG0526	Thiol-disulfide isomerase and thioredoxins	0.007	5.1E-04	2.5
57	COG0281	Malic enzyme	0.007	9.4E-05	1.3
58	COG4659	Predicted NADH:ubiquinone oxidoreductase, subunit RnfG	0.007	4.5E-05	1.1
59	COG1139	Uncharacterized conserved protein containing a ferredoxin-like domain	0.007	8.8E-05	1.8
60	COG1979	Uncharacterized oxidoreductases, Fe-dependent alcohol dehydrogenase family	0.007	1.1E-04	1.2
61	COG1146	Ferredoxin	0.008	1.7E-05	2.6
62	COG2225	Malate synthase	0.008	1.6E-05	2.5
63	COG2414	Aldehyde:ferredoxin oxidoreductase	0.009	3.3E-05	2.5
64	COG0243	Anaerobic dehydrogenases, typically selenocysteine-containing	0.009	1.2E-04	1.5
65	COG4802	Ferredoxin-thioredoxin reductase, catalytic subunit	0.009	1.7E-05	2.7
66	COG2181	Nitrate reductase gamma subunit	0.009	3.2E-05	2.5
67	COG3808	Inorganic pyrophosphatase	0.010	1.4E-04	2.0
68	COG3761	NADH:ubiquinone oxidoreductase 17.2 kD subunit	0.010	6.6E-08	Inf
69	COG4657	Predicted NADH:ubiquinone oxidoreductase, subunit RnfA	0.010	6.4E-05	1.2
70	COG4232	Thiol:disulfide interchange protein	0.010	2.7E-04	2.4
71	COG2857	Cytochrome c1	0.011	1.1E-07	5.1
72	COG4231	Indolepyruvate ferredoxin oxidoreductase, alpha and beta subunits	0.013	2.1E-04	1.8
73	COG2924	Fe-S cluster protector protein	0.014	1.0E-06	8.6
74	COG4658	Predicted NADH:ubiquinone oxidoreductase, subunit RnfD	0.014	7.2E-05	1.2
75	COG1620	L-lactate permease	0.016	4.9E-05	1.7

76	COG0667	Predicted oxidoreductases (related to aryl-alcohol dehydrogenases)	0.016	1.5E-04	1.2
77	COG1301	Na+/H+-dicarboxylate symporters	0.018	9.4E-05	1.2
78	COG2864	Cytochrome b subunit of formate dehydrogenase	0.027	1.9E-06	2.2
79	COG2086	Electron transfer flavoprotein, beta subunit	0.030	9.1E-05	1.3
80	COG0114	Fumarase	0.030	3.3E-05	1.6
81	COG3202	ATP/ADP translocase	0.031	7.5E-08	8.5
82	COG1042	Acyl-CoA synthetase (NDP forming)	0.034	5.7E-05	1.7
83	COG0633	Ferredoxin	0.034	9.2E-07	3.7
84	COG1156	Archaeal/vacuolar-type H+-ATPase subunit B	0.039	4.0E-05	1.1
85	COG1394	Archaeal/vacuolar-type H+-ATPase subunit D	0.039	4.0E-05	1.1
86	COG0377	NADH:ubiquinone oxidoreductase 20 kD subunit and related Fe-S oxidoreductases	0.039	1.0E-04	2.2
87	COG0649	NADH:ubiquinone oxidoreductase 49 kD subunit 7	0.039	1.0E-04	2.2
88	COG1005	NADH:ubiquinone oxidoreductase subunit 1 (chain H)	0.039	1.0E-04	2.2
89	COG0713	NADH:ubiquinone oxidoreductase subunit 11 or 4L (chain K)	0.039	1.0E-04	2.2
90	COG1007	NADH:ubiquinone oxidoreductase subunit 2 (chain N)	0.039	1.0E-04	2.2
91	COG0838	NADH:ubiquinone oxidoreductase subunit 3 (chain A)	0.039	1.0E-04	2.2
92	COG1008	NADH:ubiquinone oxidoreductase subunit 4 (chain M)	0.039	1.0E-04	2.2
93	COG0839	NADH:ubiquinone oxidoreductase subunit 6 (chain J)	0.039	1.0E-04	2.2

Supplementary Table 5. COGs of carbohydrate metabolism enriched in HE than H. Data were derived from two-tailed Wilcoxon test on group medians with *p* values ajusted by Benjamini-Hochberg (BH) false discovery rate for multiple testing. Difference between group medians (HE minus H) and fold change (HE divide H) were also showned. COGs were sorted in ascending order of adjusted *p* values with lexical order of COG descriptions.

#	COG ID	COG description	Adjusted *p* (BH)	HE minus H	HE divide H
1	COG1449	Alpha-amylase/alpha-mannosidase	0.004	1.7E-04	3.0
2	COG3250	Beta-galactosidase/beta-glucuronidase	0.004	1.0E-03	1.3
3	COG2376	Dihydroxyacetone kinase	0.004	1.3E-04	1.6
4	COG3594	Fucose 4-O-acetylase and related acetyltransferases	0.004	1.3E-04	1.5
5	COG0738	Fucose permease	0.004	5.2E-04	1.8
6	COG2017	Galactose mutarotase and related enzymes	0.004	2.0E-04	1.2
7	COG3408	Glycogen debranching enzyme	0.004	2.8E-04	1.6
8	COG2152	Predicted glycosylase	0.004	1.6E-04	1.5
9	COG2956	Predicted N-acetylglucosaminyl transferase	0.004	2.0E-04	3.1
10	COG3537	Putative alpha-1,2-mannosidase	0.004	1.0E-03	2.8
11	COG1877	Trehalose-6-phosphatase	0.004	6.5E-05	8.6
12	COG0380	Trehalose-6-phosphate synthase	0.004	7.3E-05	6.2
13	COG0363	6-phosphogluconolactonase/ Glucosamine-6-phosphate isomerase/deaminase	0.004	3.3E-04	1.7
14	COG3525	N-acetyl-beta-hexosaminidase	0.004	9.4E-04	2.0
15	COG0126	3-phosphoglycerate kinase	0.005	6.1E-05	1.2
16	COG0483	Archaeal fructose-1,6-bisphosphatase and related enzymes of inositol monophosphatase family	0.005	8.5E-05	1.3
17	COG2731	Beta-galactosidase, beta subunit	0.005	8.3E-05	1.3
18	COG5039	Exopolysaccharide biosynthesis protein	0.005	5.2E-05	5.2
19	COG0153	Galactokinase	0.005	7.1E-05	1.3
20	COG5026	Hexokinase	0.005	2.5E-05	5.6
21	COG0574	Phosphoenolpyruvate synthase/pyruvate phosphate dikinase	0.005	2.2E-04	1.4
22	COG0061	Predicted sugar kinase	0.005	6.1E-05	1.2
23	COG0063	Predicted sugar kinase	0.005	6.0E-05	1.2
24	COG0021	Transketolase	0.005	1.6E-04	2.4
25	COG3280	Maltooligosyl trehalose synthase	0.006	4.5E-08	2.8
26	COG0149	Triosephosphate isomerase	0.006	5.4E-05	1.1
27	COG0451	Nucleoside-diphosphate-sugar epimerases	0.006	5.3E-04	1.2
28	COG0036	Pentose-5-phosphate-3-epimerase	0.007	1.1E-04	1.3
29	COG1086	Predicted nucleoside-diphosphate sugar epimerases	0.007	2.7E-04	1.5
30	COG3717	5-keto 4-deoxyuronate isomerase	0.007	9.9E-05	1.5
31	COG3507	Beta-xylosidase	0.007	2.7E-04	1.2
32	COG0166	Glucose-6-phosphate isomerase	0.007	5.0E-05	1.1
33	COG0469	Pyruvate kinase	0.007	5.2E-05	1.1
34	COG4284	UDP-glucose pyrophosphorylase	0.007	1.7E-05	1.9
35	COG0158	Fructose-1,6-bisphosphatase	0.009	1.8E-05	2.7
36	COG0033	Phosphoglucomutase	0.009	1.6E-05	2.6
37	COG2971	Predicted N-acetylglucosamine kinase	0.009	6.5E-05	1.3
38	COG3961	Pyruvate decarboxylase and related thiamine pyrophosphate-requiring enzymes	0.009	1.7E-05	2.6
39	COG0205	6-phosphofructokinase	0.010	1.7E-04	1.2
40	COG1830	DhnA-type fructose-1,6-bisphosphate aldolase and related enzymes	0.010	1.0E-04	1.8
41	COG0837	Glucokinase	0.010	1.6E-05	2.6
42	COG0176	Transaldolase	0.010	1.8E-04	1.9
43	COG1312	D-mannonate dehydratase	0.011	6.5E-05	1.2

44	COG0148	Enolase	0.011	3.2E-05	1.1
45	COG1482	Phosphomannose isomerase	0.011	1.8E-04	1.7
46	COG0580	Glycerol uptake facilitator and related permeases (Major Intrinsic Protein Family)	0.014	3.8E-05	1.6
47	COG3936	Protein involved in polysaccharide intercellular adhesin (PIA) synthesis/biofilm formation	0.014	2.7E-08	4.4
48	COG0588	Phosphoglycerate mutase 1	0.016	4.4E-05	1.2
49	COG2942	N-acyl-D-glucosamine 2-epimerase	0.021	1.3E-04	1.7
50	COG0058	Glucan phosphorylase	0.024	3.6E-05	1.1
51	COG4975	Putative glucose uptake permease	0.024	5.6E-05	2.4
52	COG2814	Arabinose efflux permease	0.027	6.5E-04	1.4
53	COG4409	Neuraminidase (sialidase)	0.030	1.4E-04	1.8
54	COG2273	Beta-glucanase/Beta-glucan synthetase	0.034	1.3E-04	1.7
55	COG1640	4-alpha-glucanotransferase	0.044	5.3E-05	1.1
56	COG0297	Glycogen synthase	0.044	5.9E-05	1.2
57	COG0697	Permeases of the drug/metabolite transporter (DMT) superfamily	0.049	9.1E-05	1.1

Supplementary Table 6. Expression data of *Tlr4*, *Nod1* and *Nod2*. Data of gene expression are presented as $2^{-\Delta\Delta Ct}$. Three mice from the major treatment groups, H_FHE and H_FNE, and two mice from other groups were randomly selected for this experiment.

Group	Mouse ID	*Tlr4*	*Nod1*	*Nod2*
H	0401	679.7	960.1	158.9
H	0402	795.1	1146.1	117.3
HE	0201	592.4	726.3	34.5
HE	0203	464.4	628.9	39.4
H_FHE	0601	650.5	1262.7	51.3
H_FHE	0602	483.9	1002.9	59.8
H_FHE	0607	622.7	1253.4	56.6
H_FNE	0701	528.5	936.8	67.4
H_FNE	0705	644.5	1289.2	104.9
H_FNE	0706	656.1	1326.0	67.0
N	0301	490.2	634.6	50.0
N	0302	519.4	1103.4	52.2
NE	0106	302.8	652.0	30.4
NE	0107	383.2	816.6	64.3
N_FNE	0501	510.0	1041.5	89.3
N_FNE	0502	752.6	996.7	47.9

Supplementary Table 7. Magnitude of abundance changes in taxa by FMT. The magnitude of changes in *Helicobacter*, *Odoribacter*, and AF12 by FMT were qualtified by contrasting data of FMT recipient with non-FMT group at week 24 (i.e., after FMT). Data are mean relative abundance per genus and group. The last 3 columns represent the magnitude of changes in compared groups.

Genus	H	H_FHE	H_FNE	N	N_FNE	H_FHE divide H	H_FNE divide H	N_FNE divide N
**AF12**	6.7e-05	7.4e-03	8.3e-03	3.7e-03	1.7e-03	110.6	122.9	0.5
Helicobacter	1.4e-05	2.4e-02	2.6e-02	2.4e-04	9.5e-03	1732.1	1931.9	39.4
Odoribacter	1.8e-05	5.3e-02	1.7e-02	7.0e-05	4.5e-03	3001.6	938.3	63.3

Supplementary Table 8. List of primers and probes used in qPCR.

Gene	Forward primer (5' to 3')	Reverse primer (5' to 3')	UPL probe[a]
Tnf	CTgTAgCCCACgTCgTAgC	TTgAgATCCATgCCgTTg	68
Il1a	CCATAACCCATgATCTggAAG	TTggTTgAgggAATCATTCAT	29
Pparg	ggAAAgACAACggACAAATCA	ATTCggATggCCACCTCT	68
Rn18s	gCAATTATTCCCCATgAACg	gggACTTAATCAACgCAAgC	48
Tlr4	ggCAgCAggTggAATTgTAT	AggATTCgAggCTTTTCCAT	NA
Nod1	gCgAggAggTgTCTgAgTTC	gAAggggAgAAgCCAATTTC	NA
Nod2	gCTTTCTACTTggCTgTC	gTgATTTgCAggTTgTgTgg	NA
Gapdh	ACCCAgAAgACTgTggATgg	CACATTgggggTAggAACAC	NA

[a]UPL probes were purchased from Roche Molecular Biochemicals. UPL, universal probe library. NA, not applicable.

References

1 Cox, L. M. & Blaser, M. J. Antibiotics in early life and obesity. *Nat Rev Endocrinol* **11**, 182-190, doi:10.1038/nrendo.2014.210 (2015).

2 Cho, I. *et al.* Antibiotics in early life alter the murine colonic microbiome and adiposity. *Nature* **488**, 621-626, doi:10.1038/nature11400 (2012).

3 Cani, P. D. *et al.* Metabolic endotoxemia initiates obesity and insulin resistance. *Diabetes* **56**, 1761-1772, doi:10.2337/db06-1491 (2007).

4 Chi, W. *et al.* Bacterial peptidoglycan stimulates adipocyte lipolysis via NOD1. *PLoS One* **9**, e97675, doi:10.1371/journal.pone.0097675 (2014).
