# Supplementary material for: Fecal microbiota transplantation confers beneficial metabolic effects of diet and exercise on diet-induced obese mice
Source: Sci Rep. 2018 Oct 23;8:15625. doi: 10.1038/s41598-018-33893-y (PMC6199268; doi:10.1038/s41598-018-33893-y)
Supplement: Supplementary file 2 — Supplementary Dataset [file 41598_2018_33893_MOESM2_ESM.pdf]

Supplementary dataset - The R code for statistical analyses

Fecal microbiota transplantation confers beneficial metabolic effects of diet and exercise on diet-induced obese mice

Zi-Lun Lai†, Ching-Hung Tseng†, Hsiu J. Ho, Cynthia K. Y. Cheung, Jian-Yong Lin, Yi-Ju Chen, Fu-Chou Cheng, Yao-Chun Hsu, Jaw-Town Lin, Emad M. El-Omar*, Chun-Ying Wu*

†Co-first author
*Corresponding author

0. Description

This document demonstrates the R code for statistical analyses involved in the manuscript. The R code below has been arranged in the same order as the corresponding data were mentioned in the article. All data to perform the analysis below are available in Figshare (https://doi.org/10.6084/m9.figshare.5513548).

1. Food consumption and efficacy (Figure 2)

Food consumption data were recorded weekly by cage, and visualized in Figure 2A. Data were compared between non-FMT and FMT recipient groups and *t*-test was performed. Food efficacy of each group is expressed as grams of body weight gain per 100 g food consumed. Data are shown in Figure 2B.

```
library(dplyr)
library(tibble)
library(reshape2)
library(ggplot2)
library(gridExtra)

# read data
food<-read.table("foodconsumption.csv",sep=",",header=T)

# separate food and bw.gain
bwgain<-food[,c("group","bw.gain")]
food[,"bw.gain"]<-NULL

# adjust for mice number
food<-column_to_rownames(food,var="group")
food["H",]<-round(food["H",]*(6/7))
food["NE",1:7]<-round(food["NE",1:7]*(6/7))

# t test
t.test(food["N",],food["N_FNE",]) # N vs N_FNE

##
##  Welch Two Sample t-test
##
## data:  food["N", ] and food["N_FNE", ]
## t = 1.6222, df = 27.658, p-value = 0.1161
## alternative hypothesis: true difference in means is not equal to 0
## 95 percent confidence interval:
##  -2.173342 18.673342
## sample estimates:
## mean of x mean of y
##  145.1875  136.9375

t.test(food["H",],food["H_FNE",]) # H vs H_FNE

##
##  Welch Two Sample t-test
```

```
##
## data:  food["H", ] and food["H_FNE", ]
## t = -1.1832, df = 24.068, p-value = 0.2483
## alternative hypothesis: true difference in means is not equal to 0
## 95 percent confidence interval:
##  -25.725154   6.975154
## sample estimates:
## mean of x mean of y
##  112.6875  122.0625

t.test(food["H",],food["H_FHE",]) # H vs H_FHE

##
##  Welch Two Sample t-test
##
## data:  food["H", ] and food["H_FHE", ]
## t = -0.27197, df = 26.603, p-value = 0.7877
## alternative hypothesis: true difference in means is not equal to 0
## 95 percent confidence interval:
##  -11.221356   8.596356
## sample estimates:
## mean of x mean of y
##  112.6875  114.0000

# restore food table
food<-rownames_to_column(food,var="group")

# melt for ggplot
food.m<-melt(food,id.vars="group",variable.name="wk",value.name="food.consumption")

# food consumption plot
g1<-ggplot(food.m, aes(x=group, y=food.consumption)) +
  geom_boxplot(outlier.colour=NA)+
  geom_jitter(fill="grey",alpha=I(1/6),width=0.1,size=2)+
  scale_y_continuous(limits=c(0,220))+
  ggtitle("Food consumption")+
  ylab("Food consumption (g food / week)")+
  theme(axis.title.x=element_blank())

# sum up food consumption
f1<-food %>% mutate(sum=rowSums(.[2:17]))

# merge f1 with bwgain
f1<-merge(f1,bwgain,by.x="group",by.y="group")

# add n
f1$n<-7
row.h<-which(grepl("^H$",f1$group))
row.ne<-which(grepl("^NE$",f1$group))
f1[c(row.h,row.ne),'n']<-6

# food efficacy
f1<-f1 %>% mutate(f.efficacy=bw.gain*n/sum*100)

# food efficacy plot
g2<-ggplot(f1,aes(x=group,y=f.efficacy))+
  geom_bar(stat="identity",fill="white",color="black")+
  ylab("Food efficacy (gw gained / 100g food)")+
  theme(axis.title.x=element_blank())+
  ggtitle("Food efficacy")+
  geom_text(
    aes(x=group,y=f.efficacy,label=round(f.efficacy,digits=3)),
    color="black",vjust=2)

grid.arrange(g1,g2,ncol=2)
```

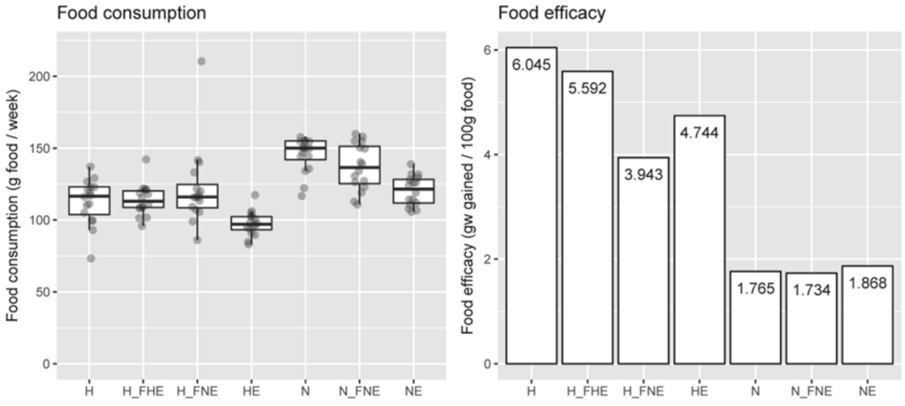

2. Physiological parameter and gene expression data (Figure 3 & Supplementary Table 3)

The body weight data for plotting Figure 3A are available as a separated file in the Figshare deposition (i.e., bodyweight.xlsx and bodyweight.csv). Two-way ANOVA was performed to test the difference in mouse body weight. The data for Figure 3B-I are presented with 5% truncated mean (data within 5–95% quantile) with standard error and available in the metadata.csv file. Student's *t*-test per group versus H was conducted for each parameter to check respective difference of means compared to H.

```
library(dplyr)
library(pipeR)
library(reshape2)

# read body weight data
bw<-read.csv(file="bodyweight.csv",stringsAsFactors=F) %>%
  melt(id.vars=c("Mouse.ID","Mouse.group"),
       maesure.vars=colnames(bw1)[-c(1,2)],
       variable.name="week",value.name="weight") %>%
  rename(group=Mouse.group,id=Mouse.ID) %>%
  mutate(week=gsub("Week\\.","",week) %>% as.numeric()) %>%
  filter(!id %in% c("H-0406","NE-0105"))

# anova
bw.fit<-aov(weight~week*group,data=bw)
summary(bw.fit)

##              Df Sum Sq Mean Sq F value Pr(>F)
## week          1   6482    6482  1043.8 <2e-16 ***
## group         6   4343     724   116.6 <2e-16 ***
## week:group    6   1315     219    35.3 <2e-16 ***
## Residuals   785   4875       6
## ---
## Signif. codes:  0 '***' 0.001 '**' 0.01 '*' 0.05 '.' 0.1 ' ' 1

# pairwise t-test
pairwise.t.test(bw$weight,bw$group,p.adjust.method="BH")

##
##  Pairwise comparisons using t tests with pooled SD
##
## data:  bw$weight and bw$group
##
##       H       H_FHE   H_FNE   HE      N       N_FNE
## H_FHE 9.2e-06 -       -       -       -       -
## H_FNE 3.8e-13 0.00265 -       -       -       -
## HE    2.9e-16 6.0e-05 0.35000 -       -       -
## N     < 2e-16 2.3e-15 6.3e-07 6.0e-05 -       -
## N_FNE < 2e-16 < 2e-16 4.2e-08 7.4e-06 0.63443 -
## NE    < 2e-16 6.1e-13 1.3e-05 0.00064 0.64058 0.36993
```

```
##
## P value adjustment method: BH

# defining function
TruncatedMean<-function(x,prop=0.05){
  x1=x[!is.na(x)]
  Lvalue=quantile(x1,prop,na.rm=T)
  Uvalue=quantile(x1,1-prop,na.rm=T)
  x2=x1[Lvalue<=x1&x1<=Uvalue]
  n=length(x2)
  return(list(N=n,Mean=mean(x2),SD=sd(x2),SE=sd(x2)/sqrt(n)))}

# read, prepare, and process data
para1<-read.csv(file="metadata.csv",stringsAsFactors=F) %>%
  filter(stage=="After") %>%
  select(c("name","group","fatw","fastglu","auc","tnfa","il1a","pparg","alt","ldl"))

para2<-melt(
  para1,id.vars=c("name","group"),
  measure.vars=c("fatw","fastglu","auc","tnfa","il1a","pparg","alt","ldl"),
  variable.name="item") %>%
    mutate(group=factor(group,levels=c("H","HE","H_FHE","H_FNE","N","NE","N_FNE")))

results<-filter(para2,!is.na(value)) %>%
  group_by(item,group) %>%
  do(k0=TruncatedMean(.$value,prop=0.05)) %>%
  summarise(Item=item,Group=group,N=k0$N,Mean=k0$Mean,SD=k0$SD,SE=k0$SE)

# display result
results

## # A tibble: 56 x 6
##       Item  Group     N    Mean          SD          SE
##     <fctr> <fctr> <int>   <dbl>       <dbl>       <dbl>
##  1    fatw      H     4   2.000  0.25245462  0.12622731
##  2    fatw     HE     5   0.946  0.10807405  0.04833218
##  3    fatw  H_FHE     5   1.298  0.36113709  0.16150542
##  4    fatw  H_FNE     5   1.076  0.55175176  0.24675089
##  5    fatw      N     5   0.332  0.07726578  0.03455431
##  6    fatw     NE     5   0.362  0.03420526  0.01529706
##  7    fatw  N_FNE     5   0.256  0.06465292  0.02891366
##  8 fastglu      H     4 155.750 10.04572878  5.02286439
##  9 fastglu     HE     5 123.400  9.83869910  4.40000000
## 10 fastglu  H_FHE     5 127.000 36.30426972 16.23576299
## # ... with 46 more rows

# t-test on truncated means
TruncatedMeanDiff<-function(x,y,prob){
  x1<-x[!is.na(x)]
  y1<-y[!is.na(x)]
  xLvalue<-quantile(x1,prob,na.rm=T)
  xUvalue<-quantile(x1,1-prob,na.rm=T)
  yLvalue<-quantile(y1,prob,na.rm=T)
  yUvalue<-quantile(y1,1-prob,na.rm=T)
  x2<-x1[xLvalue<=x1 & x1<=xUvalue]
  y2<-y1[yLvalue<=y1 & y1<=yUvalue]
  return(t.test(x2,y2)$p.value)}

# parameters to test
item<-c("fatw","fastglu","auc","tnfa","il1a","pparg","alt","ldl")

# recording p-values
diff1<-para1 %>% summarise_at(funs(TruncatedMeanDiff(.[group=="HE"],.[group=="H"],0.05)),.vars=item)
diff2<-para1 %>% summarise_at(funs(TruncatedMeanDiff(.[group=="H_FHE"],.[group=="H"],0.05)),.vars=item)
diff3<-para1 %>% summarise_at(funs(TruncatedMeanDiff(.[group=="H_FNE"],.[group=="H"],0.05)),.vars=item)
diff4<-para1 %>% summarise_at(funs(TruncatedMeanDiff(.[group=="N"],.[group=="H"],0.05)),.vars=item)
diff5<-para1 %>% summarise_at(funs(TruncatedMeanDiff(.[group=="NE"],.[group=="H"],0.05)),.vars=item)
diff6<-para1 %>% summarise_at(funs(TruncatedMeanDiff(.[group=="N_FNE"],.[group=="H"],0.05)),.vars=item)
diff0<-rbind(diff1,diff2,diff3,diff4,diff5,diff6)
diff7<-diff0 %>% apply(2,function(x)(sum(x<0.05)))
diff0<-rbind(diff0,diff7)
row.names(diff0)<-c("HE vs H","H_FHE vs H","H_FNE vs H","N vs H","NE vs H","N_FNE vs H","Sig.Count")

# display p-values and result
diff0
```

```
##                    fatw     fastglu          auc       tnfa        il1a
## HE vs H    0.0016491565 0.0022977092 0.0223446364 0.24289007 0.024602963
## H_FHE vs H 0.0112347534 0.1546420159 0.0036521231 0.06145092 0.011749075
## H_FNE vs H 0.0164034314 0.0030764680 0.0127638725 0.14116257 0.009299324
## N vs H     0.0005035731 0.0001331611 0.0002537413 0.29184366 0.299897923
## NE vs H    0.0008725018 0.0002757181 0.0001695081 0.81533302 0.503016621
## N_FNE vs H 0.0005212483 0.0003841485 0.0004695193 0.28308395 0.008566141
## Sig.Count  6.0000000000 5.0000000000 6.0000000000 0.00000000 4.000000000
##                 pparg         alt          ldl
## HE vs H    0.02053651 0.006060989 5.856668e-04
## H_FHE vs H 0.12772820 0.004826945 4.437725e-04
## H_FNE vs H 0.49593882 0.007200505 1.064144e-04
## N vs H     0.31792543 0.001902208 2.747071e-06
## NE vs H    0.60071074 0.002320047 2.801869e-05
## N_FNE vs H 0.08176965 0.002325832 4.866186e-06
## Sig.Count  1.00000000 6.000000000 6.000000e+00
```

3. Principal coordinates analysis (Figure 4)

Principal coordinates analysis (PCoA) was conducted based on Bray–Curtis distance of OTU relative abundance in mice gut microbiota. Mice groups are distinguished by colors. The significance (*p* value) of between-group inertia was tested by Monte-Carlo test (with 1,000 permutations).

```
library(ade4)
library(dplyr)
library(vegan)
library(ggplot2)
library(phyloseq)

# read and prepare metadata
metadata<-read.csv(file="metadata.csv",stringsAsFactors=T) %>%
  select(c("name","stage","group"))
rownames(metadata)<-metadata$name

metadata.bef<-metadata %>% filter(stage=="Before")
rownames(metadata.bef)<-metadata.bef$name

metadata.aft<-metadata %>% filter(stage=="After")
rownames(metadata.aft)<-metadata.aft$name

# read and process otu table
otu.tbl<-read.csv(file="otu.abundance.csv",stringsAsFactors=F,row.names=1)
colnames(otu.tbl)<-gsub("\\.","-",colnames(otu.tbl))

otu.tbl.bef<-otu.tbl %>% select(metadata.bef$name)
otu.tbl.aft<-otu.tbl %>% select(metadata.aft$name)

# create phyloseq object
phylo.bef<-phyloseq(
  otu_table(otu.tbl.bef,taxa_are_rows=T),
  sample_data(metadata.bef)) %>%
  transform_sample_counts(function(x)x/sum(x))
phylo.aft<-phyloseq(
  otu_table(otu.tbl.aft,taxa_are_rows=T),
  sample_data(metadata.aft)) %>%
  transform_sample_counts(function(x)x/sum(x))

# PCoA (before FMT)
pcoa.class.bef<-metadata.bef$group
pcoa.res.bef<-distance(phylo.bef,"bray") %>% dudi.pco(scannf=F)

# Monte-Carlo test (before FMT)
rtest(bca(pcoa.res.bef,pcoa.class.bef,scan=FALSE),nrepet=1000)

## Monte-Carlo test
## Call: rtest.between(xtest = bca(pcoa.res.bef, pcoa.class.bef, scan = FALSE),
##     nrepet = 1000)
##
## Observation: 0.6139047
##
## Based on 1000 replicates
## Simulated p-value: 0.000999001
```

```
## Alternative hypothesis: greater
##
##      Std.Obs  Expectation     Variance
## 18.066015272  0.130010270  0.000717425

# process PCoA plot (before FMT)
ord1<-ordinate(phylo.bef,"PCoA","bray")
ord2<-plot_ordination(phylo.bef,ord1,justDF=T)[,1:2]
ord3<-ordiellipse(ord2,pcoa.class.bef,kind="sd",conf=0.95,draw="none")

# ellipse (before FMT)
df_ell<-data.frame()
for(g in names(ord3)){
  df_ell<-rbind(df_ell,data.frame(
    vegan:::veganCovEllipse(cov=ord3[[g]]$cov,center=ord3[[g]]$center,
                            scale=ord3[[g]]$scale),group=g))}
colnames(df_ell)=c("Axis.1","Axis.2","label")

# spider (before FMT)
muS=by(ord2,pcoa.class.bef,function(x){list(mu=colMeans(x),S=cov(x))})
spider=lapply(1:nsamples(phylo.bef),function(x){c(ord2[x,1:2],muS[[pcoa.class.bef[x]]]$mu)}) %>%
  do.call(rbind,.) %>% data.frame(stringsAsFactors=F)
for(i in 1:4){spider[[i]]=unlist(spider[[i]])}
spider$label=pcoa.class.bef

# plot PCoA (before FMT)
group.color<-c("#F06BA8","#ED1C24","#CE2090","#EE7F62","#66A9DD","#3850A2","#7A80BB")
pcoa.plot.bef<-plot_ordination(phylo.bef,ord1,type="samples",color="group") +
  geom_path(data=df_ell,aes(x=Axis.1,y=Axis.2,colour=label),size=1,linetype=1) +
  geom_segment(mapping=aes(x=Axis.1,y=Axis.2,xend=Axis.1.1,yend=Axis.2.1,colour=label),data=spider) +
  scale_colour_manual(values=group.color) +
  annotate("text",x=-0.45,y=-0.5,label="P<0.001") +
  ggtitle("Before FMT (week 12)") +
  theme(plot.title=element_text(hjust=0.5))

# display PCoA (before FMT)
pcoa.plot.bef
```

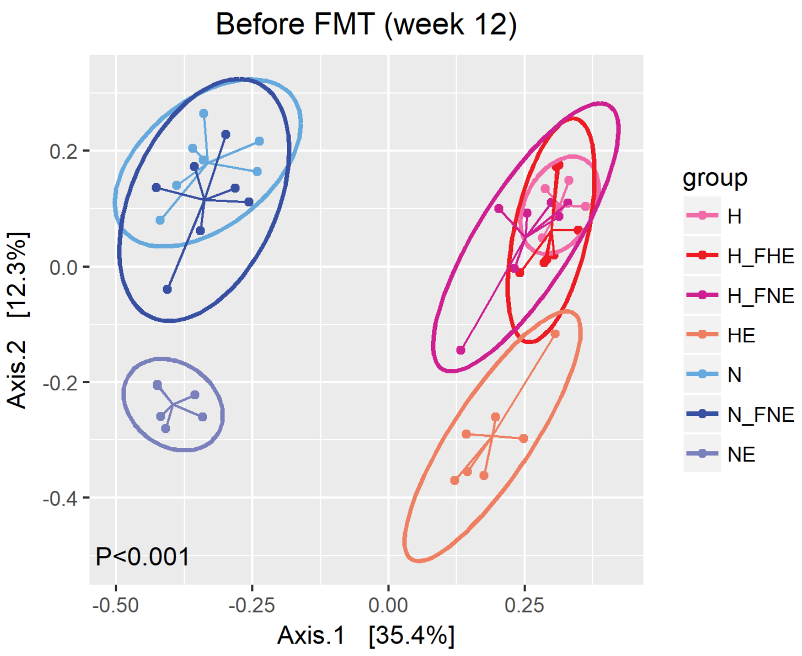


```
# PCoA (after FMT)
pcoa.class.aft<-metadata.aft$group
pcoa.res.aft<-distance(phylo.aft,"bray") %>% dudi.pco(scannf=F)

# Monte-Carlo test (after FMT)
rtest(bca(pcoa.res.aft,pcoa.class.aft,scan=FALSE),nrepet=1000)
```

```
## Monte-Carlo test
## Call: rtest.between(xtest = bca(pcoa.res.aft, pcoa.class.aft, scan = FALSE),
##     nrepet = 1000)
##
## Observation: 0.6003946
##
## Based on 1000 replicates
## Simulated p-value: 0.000999001
## Alternative hypothesis: greater
##
##      Std.Obs  Expectation     Variance
## 1.744702e+01 1.304790e-01 7.254327e-04

# process PCoA plot (after FMT)
ord1<-ordinate(phylo.aft,"PCoA","bray")
ord2<-plot_ordination(phylo.aft,ord1,justDF=T)[,1:2]
ord3<-ordiellipse(ord2,pcoa.class.aft,kind="sd",conf=0.95,draw="none")

# ellipse (after FMT)
df_ell<-data.frame()
for(g in names(ord3)){
  df_ell<-rbind(df_ell,data.frame(
    vegan:::veganCovEllipse(cov=ord3[[g]]$cov,center=ord3[[g]]$center,
                            scale=ord3[[g]]$scale),group=g))}
colnames(df_ell)=c("Axis.1","Axis.2","label")

# spider (after FMT)
muS=by(ord2,pcoa.class.aft,function(x){list(mu=colMeans(x),S=cov(x))})
spider=lapply(1:nsamples(phylo.aft),function(x){c(ord2[x,1:2],muS[[pcoa.class.aft[x]]]$mu)}) %>%
  do.call(rbind,.) %>% data.frame(stringsAsFactors=F)
for(i in 1:4){spider[[i]]=unlist(spider[[i]])}
spider$label=pcoa.class.aft

# plot PCoA (after FMT)
group.color<-c("#F06BA8","#ED1C24","#CE2090","#EE7F62","#66A9DD","#3850A2","#7A80BB")
pcoa.plot.aft<-plot_ordination(phylo.aft,ord1,type="samples",color="group") +
  geom_path(data=df_ell,aes(x=Axis.1,y=Axis.2,colour=label),size=1,linetype=1) +
  geom_segment(mapping=aes(x=Axis.1,y=Axis.2,xend=Axis.1.1,yend=Axis.2.1,colour=label),data=spider) +
  scale_colour_manual(values=group.color) +
  annotate("text",x=-0.5,y=-0.45,label="P<0.001") +
  ggtitle("After FMT (week 24)") +
  theme(plot.title=element_text(hjust=0.5))

# display PCoA (after FMT)
pcoa.plot.aft
```

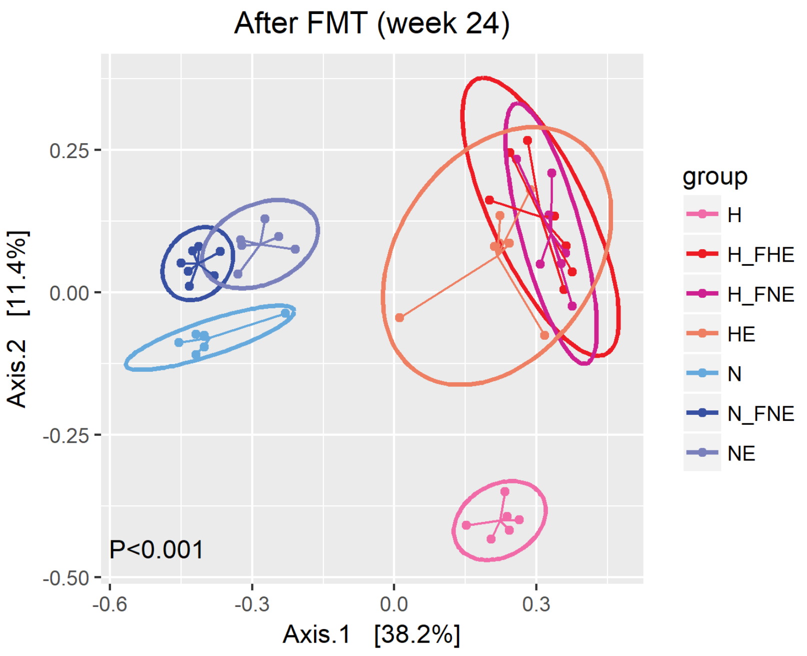

4. Two-way PERMANOVA test

Two-way PERMANOVA was performed to quantitatively compare the influences of diet and exercise on determining gut microbiota. As with default, PERMANOVA was tested with 999 permutations.

```
library(vegan)

# read metadata
metadata<-read.csv(file="metadata.csv",stringsAsFactors=T) %>%
  filter(stage=="Before") %>%
  select(c("name","stage","group","diet","exercise"))

# read and process otu table
otu.tbl<-read.csv(file="otu.abundance.csv",stringsAsFactors=F,row.names=1)
colnames(otu.tbl)<-gsub("\\.","-",colnames(otu.tbl))

# select samples before FMT and calculate distance matrix
otu.tbl.before<-otu.tbl %>% select(metadata$name)
dist.mx<-vegdist(t(otu.tbl.before),method="bray")

# Two-way PERMANOVA test
adonis(dist.mx~diet+exercise,data=metadata)


##
## Call:
## adonis(formula = dist.mx ~ diet + exercise, data = metadata)
##
## Permutation: free
## Number of permutations: 999
##
## Terms added sequentially (first to last)
##
##           Df SumsOfSqs MeanSqs F.Model      R2 Pr(>F)
## diet       1    4.0345  4.0345 21.5446 0.29664  0.001 ***
## exercise   1    1.3266  1.3266  7.0842 0.09754  0.001 ***
## Residuals 44    8.2395  0.1873         0.60582
## Total     46   13.6006                 1.00000
## ---
## Signif. codes:  0 '***' 0.001 '**' 0.01 '*' 0.05 '.' 0.1 ' ' 1
```

5. Interaction analysis (Supplementary Figure 4)

This analysis explores and visualizes interaction between the effects of two factors. In the manuscript, diet vs. Shannon and exercise vs. Shannon were analyzed.

```
library(dplyr)
library(ggplot2)
library(phyloseq)
library(gridExtra)

# read metadata
metadata<-read.csv(file="metadata.csv",stringsAsFactors=F) %>%
  select(c("name","stage","group","diet","exercise","fmt"))
metadata$exercise<-gsub("non-exercise","None",metadata$exercise)
metadata$exercise<-gsub("exercise","Exec",metadata$exercise)
rownames(metadata)<-metadata$name

# read and process otu table
otu.tbl<-read.csv(file="otu.abundance.csv",stringsAsFactors=F,row.names=1)
colnames(otu.tbl)<-gsub("\\.","-",colnames(otu.tbl))

# create phyloseq object
phylo<-phyloseq(
  otu_table(otu.tbl,taxa_are_rows=T),
  sample_data(metadata))

# shannon index
div.idx<-estimate_richness(phylo,measures=c("Shannon"))
div.idx<-merge(x=metadata,y=div.idx,by.x="name",by.y=0)

# interaction plot
```

```
div.idx<-div.idx %>% filter(fmt=="non-fmt")
shannon.summ<-plyr::ddply(div.idx,plyr::.(exercise,diet),summarise,val=mean(Shannon))

# diet vs shannon
g1<-ggplot(div.idx,aes(x=diet,y=Shannon,colour=exercise))+
  geom_boxplot(outlier.colour=NA)+
  geom_jitter(position=position_jitter(width=.5,height=0),alpha=0.3)+
  geom_point(data=shannon.summ,aes(y=val))+
  geom_line(data=shannon.summ,aes(y=val,group=exercise))

# exercise vs shannon
g2<-ggplot(div.idx,aes(x=exercise,y=Shannon,colour=diet))+
  geom_boxplot(outlier.colour=NA)+
  geom_jitter(position=position_jitter(width=.5,height=0),alpha=0.3)+
  geom_point(data=shannon.summ,aes(y=val))+
  geom_line(data=shannon.summ,aes(y=val,group=diet))

# display interaction plots
grid.arrange(g1,g2,ncol=2)
```

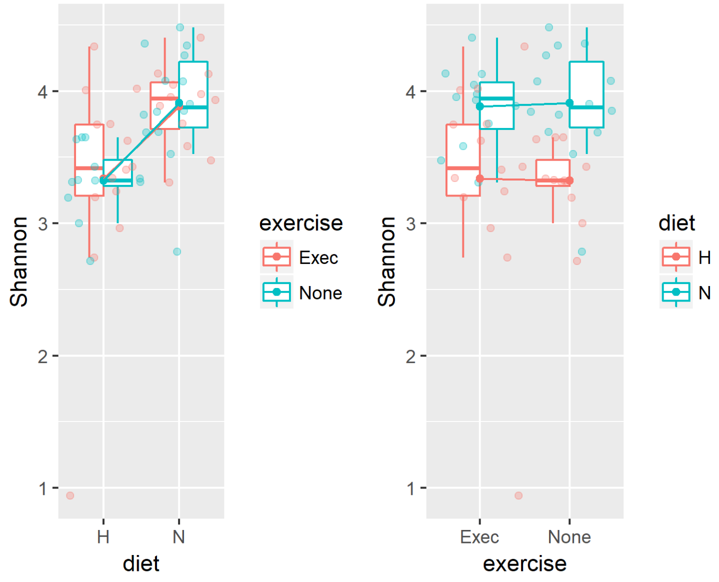

6. LEfSe analysis (Figure 5)

LEfSe analysis of genus profile was performed to identify genera with contrasting relative abundances between the given two groups. In the manuscript seven comparisons were reported in Figure 5, including (1) H vs. N, (2) HE vs. NE, (3) H vs. HE, (4) N vs. NE, (5) H vs. H_FHE, (6) H vs. H_FNE, and (7) N vs. N_FNE. The following code demonstrates "H vs. N" to exemplify the analytic flow. Other comparisons can be repeated using by just substituting group names in the code below.

Enriched genera reported by LEfSe were further filtered by a group-mean difference in relative abundance larger than 10^{-5} to exclude genera of absence in one group and minute abundance in the other. To deliver a more intuitive perception of contrasting abundance, LEfSe results were visualized by plotting the log-2 ratio of average relative abundance between groups (i.e., log2diff in the R results below) rather than LDA score.

```
library(dplyr)
library(pipeR)
library(tibble)
library(phyloseq)
```

```r
# read metadata
metadata<-read.csv(file="metadata.csv",stringsAsFactors=F) %>%
  select(c("name","stage","group"))
rownames(metadata)<-metadata$name

# read and process otu table
otu.tbl<-read.csv(file="otu.abundance.csv",stringsAsFactors=F,row.names=1)
colnames(otu.tbl)<-gsub("\\.","-",colnames(otu.tbl))

# read tax table
tax.tbl<-read.csv(file="otu.taxonomy.csv",stringsAsFactors=F,row.names=1)

# create phyloseq object
phylo<-phyloseq(
  otu_table(otu.tbl,taxa_are_rows=T),
  tax_table(as.matrix(tax.tbl)),
  sample_data(metadata))

# create % genus profile for LEfSe
phylo.gen<-tax_glom(phylo,taxrank="Genus",NArm=F) %>%
  transform_sample_counts(function(x)x/sum(x))

# H vs N
sample.keep<-metadata %>% filter(stage=="After" & group %in% c("H","N")) %>% select("name")
phylo.gen.tmp<-prune_samples(sample.keep$name,phylo.gen)

a1<-sample_data(phylo.gen.tmp) %>% data.frame() %>% select(c("name","group"))
a2<-otu_table(phylo.gen.tmp) %>% t() %>% as.data.frame()
a3<-merge(x=a1,y=a2,by.x="name",by.y=0) %>% t()
a4<-data.frame("genus"=apply(tax_table(phylo.gen.tmp)@.Data[,1:6],1,paste,collapse="|"))
dummyrow<-data.frame(genus=c("name","group"),row.names=c("name","group"))
a5<-rbind(dummyrow,a4) %>%
  rownames_to_column(var="ggid") %>%
  filter(!grepl("\\|$",genus,perl=T)) %>%
  column_to_rownames('ggid')
a6<-merge(x=a5,y=a3,by.x=0,by.y=0,sort=F)

write.table(a6[-1,-1],file="lefse.txt",sep="\t",quote=F,row.names=F,col.names=F)

# perform lefse outside R
system("path/to/python format_input.py lefse.txt lefse.in -c 1 -s -1 -u -1 -o 1000000",intern =T)
system("path/to/python run_lefse.py lefse.in lefse.res -y 0",intern =T)

# read lefse data
b0<-read.delim("lefse.res",header=F,stringsAsFactors=F)
b1<-read.delim("lefse.txt",header=F,stringsAsFactors=F)

# significant taxa by LEfSe
b01<-b0 %>% filter(V3!="")

# process genus name
# (1) pipe to dot; (2) space to none; (3) square brackets & dash to underscore
b11<-mutate(b1,nv1=gsub("\\|","\\.",V1) %>>% (gsub("\\s","",.)) %>>% (gsub("\\[|\\]|-","_",.))) %>>%
  filter(nv1=="group"|nv1%in%b01$V1)

# grouped average relative abundance
b12<-b11 %>% select(-V1,-nv1)
rownames(b12)<-b11$V1

b13<-sapply(2:nrow(b12),function(i){
  split(as.numeric(unlist(b12[i,])),unlist(b12["group",])) %>>% sapply(mean)
  }) %>>% t() %>>%
  data.frame(Genus=sub(".*\\|","",b11$V1[-1]),stringsAsFactors=F) %>>%
  mutate(absdiff=abs(H-N),log2diff=log2(H/N)) %>>%
  filter(absdiff>0.00001&!is.infinite(log2diff)) %>>%
  arrange(desc(log2diff))

# list top-5 genera with largest difference on relative abundance per group
bind_rows(
  filter(b13,log2diff>0) %>>% top_n(5,log2diff),
  filter(b13,log2diff<0) %>>% top_n(5,-log2diff))

##               H            N         Genus      absdiff  log2diff
## 1  6.689052e-04 1.757550e-06   Lactococcus 6.671477e-04  8.572092
## 2  2.634976e-02 3.618960e-04 Lactobacillus 2.598786e-02  6.186071
## 3  7.915286e-05 1.760973e-06 Anaerotruncus 7.739188e-05  5.490197
## 4  7.109703e-05 1.821831e-06         SMB53 6.927520e-05  5.286328
```

```
## 5  4.566071e-04 4.100972e-05     Bilophila 4.155974e-04  3.476916
## 6  1.363948e-05 2.405595e-04  Helicobacter 2.269200e-04 -4.140533
## 7  6.717498e-05 3.669354e-03          AF12 3.602179e-03 -5.771458
## 8  7.701929e-04 6.026970e-02    Prevotella 5.949951e-02 -6.290069
## 9  5.751620e-05 4.605340e-03    Sutterella 4.547824e-03 -6.323196
## 10 2.075732e-06 5.791605e-04  Turicibacter 5.770848e-04 -8.124199
```

7. Functional enrichment analysis (Supplementary Table 4 & 5)

The functional enrichment analysis was performed on the COGs profile predicted by using PICRUSt.

```
options(width=120)
library(dplyr)
library(reshape2)
library(phyloseq)

# read metadata
metadata<-read.csv(file="metadata.csv",stringsAsFactors=F) %>%
  select(c("name","stage","group"))
rownames(metadata)<-metadata$name

# process metadata
metadata$beforeG<-metadata$group
metadata$beforeG[grepl("^N_",metadata$group,perl=T)]<-"N"
metadata$beforeG[grepl("^H_",metadata$group,perl=T)]<-"H"
metadata$afterG<-metadata$group

# read and process COG abundance table
cog.tbl<-read.csv(file="picrust.cog.abundance.csv",stringsAsFactors=F,row.names=1)
colnames(cog.tbl)<-gsub("\\.","-",colnames(cog.tbl))

# read COG annotation table
cog.anno.tbl<-read.csv(file="picrust.cog.annotation.csv",stringsAsFactors=F,row.names=1)

# create phyloseq object for COGs
phylo.cog<-phyloseq(
  otu_table(cog.tbl,taxa_are_rows=T),
  tax_table(as.matrix(cog.anno.tbl)),
  sample_data(metadata))

# prepare COGs phyloseq object for later analysis
phylo.cogr<-transform_sample_counts(phylo.cog,function(x)x/sum(x)) %>% psmelt()
names(phylo.cogr)[1]<-"COG.Id"

# prepare COG profile (before FMT)
before.cog<-phylo.cogr %>% filter(stage=='Before' & beforeG %in% c('H','HE'))

# summarise COG median by group
c0<-before.cog %>%
  group_by(COG.Id,beforeG) %>%
  summarise(Median=median(Abundance)) %>%
  data.table::dcast(COG.Id~beforeG,value.var="Median") %>%
  setNames(c("COG.Id","H.median","HE.median"))

# wx.test per COG, H vs HE
c1<-before.cog %>%
  filter(beforeG %in% c('H','HE')) %>%
  group_by(COG.Id) %>%
  do(k0=wilcox.test(Abundance~beforeG,data=.,exact=FALSE)) %>%
  summarise(COG.Id,pvalue=k0$p.value) %>%
  setNames(c("COG.Id","H.HE.wx.p")) %>% as.data.frame()

# merge median and wx.test p-value
c0<-merge(x=c0,y=c1,by.x="COG.Id",by.y="COG.Id")

# p-value adjustment (BH) for multiple testing
c0<-c0 %>% mutate(
  HE.minus.H=HE.median-H.median,
  HE.divide.H=HE.median/H.median,
  H.HE.wx.p.adj=p.adjust(H.HE.wx.p,"BH"))

# display partial results
head(c0)
```

```
##    COG.Id     H.median    HE.median    H.HE.wx.p    HE.minus.H HE.divide.H H.HE.wx.p.adj
## 1 COG0001 2.277205e-04 1.081453e-04 0.0006672533 -1.195752e-04   0.4749036   0.005031790
## 2 COG0002 3.691179e-04 4.172695e-04 0.0025664548  4.815166e-05   1.1304506   0.008902972
## 3 COG0003 3.398688e-05 4.412653e-05 0.1281270965  1.013965e-05   1.2983402   0.194769474
## 4 COG0004 3.340386e-04 2.553625e-04 0.0003587877 -7.867608e-05   0.7644701   0.004477047
## 5 COG0005 3.428015e-04 4.104000e-04 0.0008158396  6.759851e-05   1.1971943   0.005031790
## 6 COG0006 7.143192e-04 9.846670e-04 0.0012091728  2.703479e-04   1.3784693   0.006174957

# filter COGs of significance
significant.cogid<-c0 %>% filter(H.HE.wx.p.adj<0.05)

# select COGs of Energy category [C]
energy.cog<-before.cog %>%
  filter(COG.Id %in% significant.cogid$COG.Id & grepl("\\[C\\]",Category)) %>%
  select(COG.Id,Description) %>% unique()

# filter COGs enriched in HE group
energy.cog.HE<-c0 %>%
  filter(COG.Id %in% energy.cog$COG.Id & HE.minus.H>0) %>%
  select(COG.Id,H.HE.wx.p.adj,HE.minus.H,HE.divide.H) %>%
  arrange(H.HE.wx.p.adj)

# arrange output
energy.cog.HE<-merge(x=energy.cog.HE,y=energy.cog,by.x="COG.Id",by.y="COG.Id",all.x=TRUE)
energy.cog.HE<-energy.cog.HE[,c(1,5,2:4)] %>%
  arrange(Description) %>% arrange(H.HE.wx.p.adj)

# number of significant COGs
nrow(energy.cog.HE)

## [1] 93

# display partial results
head(energy.cog.HE)

##    COG.Id                                                                       Description H.HE.wx.p.adj
## 1 COG1271                                         Cytochrome bd-type quinol oxidase, subunit 1   0.004107806
## 2 COG1294                                         Cytochrome bd-type quinol oxidase, subunit 2   0.004107806
## 3 COG0712 F0F1-type ATP synthase, delta subunit (mitochondrial oligomycin sensitivity protein)   0.004107806
## 4 COG0247                                                                 Fe-S oxidoreductase   0.004107806
## 5 COG0731                                                                Fe-S oxidoreductases   0.004107806
## 6 COG1148                     Heterodisulfide reductase, subunit A and related polyferredoxins   0.004107806
##     HE.minus.H HE.divide.H
## 1 0.0001945352    2.929677
## 2 0.0001739717    2.929584
## 3 0.0001041576    1.341763
## 4 0.0003032202    2.514077
## 5 0.0001722983    2.987090
## 6 0.0001218479    2.680070

# select COGs of Carbohydrate category [G]
carb.cog<-before.cog %>%
  filter(COG.Id %in% significant.cogid$COG.Id & grepl("\\[G\\]",Category)) %>%
  select(COG.Id,Description) %>% unique()

# filter COGs enriched in HE group
carb.cog.HE<-c0 %>%
  filter(COG.Id %in% carb.cog$COG.Id & HE.minus.H>0) %>%
  select(COG.Id,H.HE.wx.p.adj,HE.minus.H,HE.divide.H) %>%
  arrange(H.HE.wx.p.adj)

# arrange output
carb.cog.HE<-merge(x=carb.cog.HE,y=carb.cog,by.x="COG.Id",by.y="COG.Id",all.x=TRUE)
carb.cog.HE<-carb.cog.HE[,c(1,5,2:4)] %>%
  arrange(Description) %>% arrange(H.HE.wx.p.adj)

# number of significant COGs
nrow(carb.cog.HE)

## [1] 57

# display partial results
head(carb.cog.HE)

##    COG.Id                                 Description H.HE.wx.p.adj   HE.minus.H HE.divide.H
## 1 COG1449                     Alpha-amylase/alpha-mannosidase   0.004107806 0.0001708345    2.987800
```

```
## 2 COG3250               Beta-galactosidase/beta-glucuronidase   0.004107806 0.0010269648    1.346889
## 3 COG2376                              Dihydroxyacetone kinase   0.004107806 0.0001300251    1.553818
## 4 COG3594 Fucose 4-O-acetylase and related acetyltransferases   0.004107806 0.0001278937    1.451708
## 5 COG0738                                      Fucose permease   0.004107806 0.0005199937    1.770405
## 6 COG2017            Galactose mutarotase and related enzymes   0.004107806 0.0001954167    1.248804
```

8. Magnitude of abundance changes in taxa by FMT (Supplementary Table 7)

The magnitude of changes in *Helicobacter*, *Odoribacter* and AF12 by FMT were qualtified by contrasting data of FMT recipient with non-FMT group at week 24 (i.e., after FMT).

```
options(width=90)
library(dplyr)
library(reshape2)
library(phyloseq)

# read metadata
metadata<-read.csv(file="metadata.csv",stringsAsFactors=F) %>%
  select(c("name","stage","group"))
rownames(metadata)<-metadata$name

# read and process otu table
otu.tbl<-read.csv(file="otu.abundance.csv",stringsAsFactors=F,row.names=1)
colnames(otu.tbl)<-gsub("\\.","-",colnames(otu.tbl))

# read tax table
tax.tbl<-read.csv(file="otu.taxonomy.csv",stringsAsFactors=F,row.names=1)

# create phyloseq object
phylo<-phyloseq(
  otu_table(otu.tbl,taxa_are_rows=T),
  tax_table(as.matrix(tax.tbl)),
  sample_data(metadata))

# create % genus profile for LEfSe
phylo.gen<-tax_glom(phylo,taxrank="Genus",NArm=F) %>%
  transform_sample_counts(function(x)x/sum(x))

# subset samples after FMT and H/N/H_FHE/H_FNE/N_FNE
a1<-subset_samples(phylo.gen,stage=="After" & group %in% c("H","N","H_FHE","H_FNE","N_FNE"))

# row index of Helicobacter/Odoribacter/AF12
row.heli<-which(tax_table(a1)[,"Genus"]=="Helicobacter")
row.odor<-which(tax_table(a1)[,"Genus"]=="Odoribacter")
row.af12<-which(tax_table(a1)[,"Genus"]=="AF12")

# process magnitude
magnitude<-a1 %>% psmelt() %>%
  filter(Genus %in% c("Helicobacter","Odoribacter","AF12")) %>%
  group_by(Genus,group) %>%
  summarise(AvgRelAbd=mean(Abundance)) %>%
  dcast(Genus~group,value.var="AvgRelAbd") %>%
  mutate(H_FHE.divide.H=H_FHE/H,
         H_FNE.divide.H=H_FNE/H,
         N_FNE.divide.N=N_FNE/N)

# display results
magnitude

##          Genus            H       H_FHE       H_FNE            N       N_FNE
## 1         AF12 6.717498e-05 0.007429469 0.008257138 3.669354e-03 0.001682154
## 2 Helicobacter 1.363948e-05 0.023624602 0.026349714 2.405595e-04 0.009489210
## 3  Odoribacter 1.766371e-05 0.053019243 0.016573156 7.047669e-05 0.004464555
##   H_FHE.divide.H H_FNE.divide.H N_FNE.divide.N
## 1       110.5988       122.9198      0.4584332
## 2      1732.0749      1931.8707     39.4464158
## 3      3001.5917       938.2602     63.3479622
```
